# Interweaving Insights: High-Order Feature Interaction for Fine-Grained Visual Recognition

**DOI:** 10.1007/s11263-024-02260-y

**Published:** 2024-10-20

**Authors:** Arindam Sikdar, Yonghuai Liu, Siddhardha Kedarisetty, Yitian Zhao, Amr Ahmed, Ardhendu Behera

**Affiliations:** 1 Department of Computer Science, Edge Hill University, Ormskirk, UK; 2 Department of Aerospace Engineering, Technion—Israel Institute of Technology, Haifa, Israel; 3 Ningbo Institute of Materials Technology and Engineering, Chinese Academy of Sciences, Ningbo, China

**Keywords:** Inter-region and intra-region graphs, Convolutional neural networks, Residual graph neural networks, Graph attention networks, Fine-grained visual recognition, High-order feature interaction

## Abstract

This paper presents a novel approach for Fine-Grained Visual Classification (FGVC) by exploring Graph Neural Networks (GNNs) to facilitate high-order feature interactions, with a specific focus on constructing both inter- and intra-region graphs. Unlike previous FGVC techniques that often isolate global and local features, our method combines both features seamlessly during learning via graphs. Inter-region graphs capture long-range dependencies to recognize global patterns, while intra-region graphs delve into finer details within specific regions of an object by exploring high-dimensional convolutional features. A key innovation is the use of shared GNNs with an attention mechanism coupled with the Approximate Personalized Propagation of Neural Predictions (APPNP) message-passing algorithm, enhancing information propagation efficiency for better discriminability and simplifying the model architecture for computational efficiency. Additionally, the introduction of residual connections improves performance and training stability. Comprehensive experiments showcase state-of-the-art results on benchmark FGVC datasets, affirming the efficacy of our approach. This work underscores the potential of GNN in modeling high-level feature interactions, distinguishing it from previous FGVC methods that typically focus on singular aspects of feature representation. Our source code is available at https://github.com/Arindam-1991/I2-HOFI.

## Introduction

The ascendancy of deep Convolutional Neural Networks (CNNs) has markedly improved image recognition over the last decade. This progress is chiefly ascribed to their proficiency in offering a high-level representation, encompassing global shape and appearance, by extracting pertinent object-pose and parts information from texture and shape. Such representations are particularly effective for Large-Scale Visual Classification (LSVC) tasks involving distinct categories, as evidenced by their success on datasets like ImageNet and COCO. However, when it comes to addressing Fine-Grained Visual Classification (FGVC) challenges, CNNs exhibit limitations compared to their LSVC prowess. This disparity stems from the intricate nature of subtle distinctions between object classes in FGVC, nuances that are challenging for the model to discern but often perceptible to human observers. This is primarily attributed to the presence of subordinate object categories within the same entry-level category, encompassing distinctions like bird species (Wah et al., 2011), dog breeds (Khosla et al., 2011), various types of flowers (Nilsback & Zisserman, 2008), as well as man-made objects including car models (Krause et al., 2013), aircraft types (Maji et al., 2013) and more. An inherent observation in such datasets reveals that disparate classes often share analogous visual structures (resulting in significant inter-class resemblances), while items within the same class frequently display notable disparities due to differing structures, lighting conditions, clutter, and viewpoints (leading to substantial intra-class variations). Consequently, acquiring a unified and distinguishing representation for each class poses a formidable challenge. A crucial step in tackling this challenge involves extracting distinct features from essential object parts and fusing them to represent a consistently unique overall structure for a specific class. Contemporary state-of-the-art (SotA) methods are cleverly designed to extract these discriminative features and structures by either utilizing human-provided part annotations or autonomously discovering such discriminative parts within the entire image. For a comprehensive survey, readers can refer to Wei et al. (2021). Previous efforts primarily fall into the first category, relying on annotations like bounding boxes or masks for pinpointing discriminative object parts. Some approaches involve training part-based detectors, while the others opt for semantic segmentation to localize parts. Nevertheless, these part annotations demand substantial labor, are susceptible to errors, and necessitate domain expertise. Additionally, part-based approaches constrain scalability and practicality in real-world FGVC tasks. Hence, this led to recent techniques leveraging image-level labels to guide models in discerning key object parts for sub-category discrimination by employing attention mechanisms within image or feature spaces (Behera et al., 2021, 2020; Liu et al., 2019; Bera et al., 2021), automatically revealing discriminative features.

**Fig. 1:**
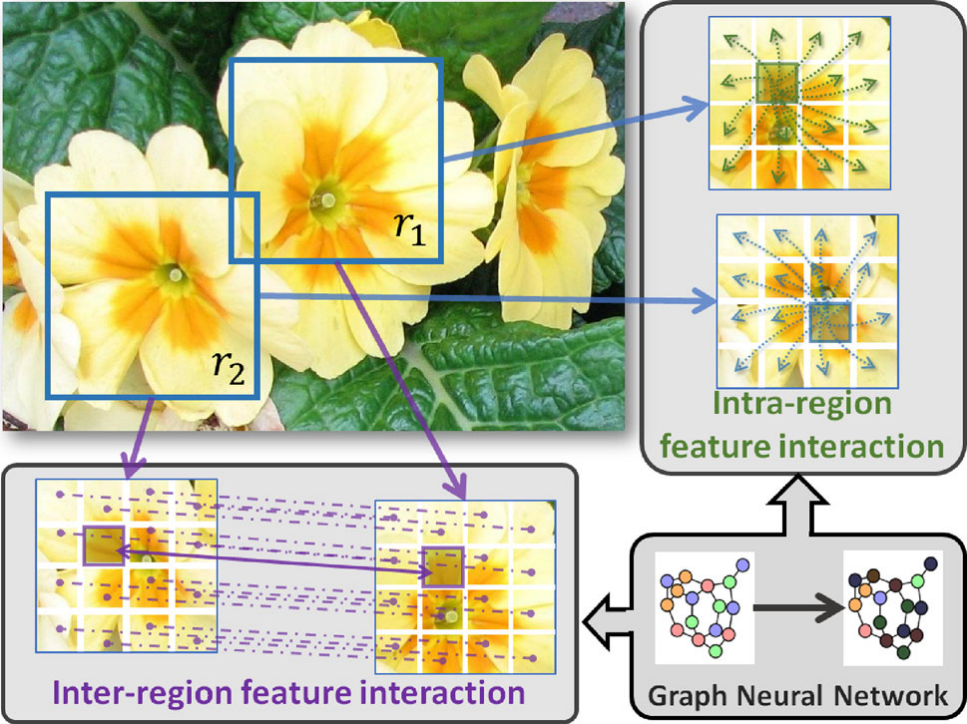
We introduce a novel approach for inter- and intra-region high-order feature interaction, aimed at capturing intricate relationships among object parts. This enhances discriminability by effectively addressing subtle variations within the context of Fine-Grained Visual Classification (FGVC)

### Motivation

In this research, we tackle the challenge of mining fine-grained information for object recognition through two key innovations as shown in Fig. 1. Firstly, we focus on establishing inter-relationships among multiple parts of the same object, capturing long-range dependencies often perceived as disjointed features by conventional convolutional filters. Secondly, we aim to enhance the informativeness of specific regions within objects, crucial for adding discriminative power essential in FGVC.

Various part-based models (Huang et al., 2016; Zhang et al., 2016; Liu et al., 2021) for FGVC necessitate dense part annotations, involving techniques like detected keypoints (Bera et al., 2021), pose normalization (Simon et al., 2020), part-level bounding boxes with R-CNN models (Zhang et al., 2014), and linkage functions (Lin et al., 2020). Some models (Yu et al., 2018; Chai et al., 2013) incorporate segmentation to enhance localization accuracy. However, these annotation-intensive processes hinder scalability and practicality in real-world applications. Models relying on part-based annotations may overlook valuable information beyond predefined boundaries, and the discrete definition of specific parts in fine-grained recognition restricts the investigation of additional object regions that may contain essential discriminative information. In contrast, ‘weakly-supervised’ methods (Zhang et al., 2016) use image-level labels to identify semantic groups for fine-grained recognition, employing techniques like spatial pyramids (Yan et al., 2017), part proposal clustering (He et al., 2023), and co-segmentation (Hung et al., 2019). However, they face challenges related to localization ambiguity and bridging the semantic gap between image-level labels and fine-grained attributes or parts, such as distinguishing aircraft with similar body colors and nearly identical shapes but differing wing designs.

In practice, generating a sufficient number of labels, whether they be part annotations or weak labels, to distinctly identify fine-grained objects within the same broader class poses a challenge for human annotators, especially when differences are minimal (Wei et al., 2019). This labor-intensive labeling process becomes a significant bottleneck in developing fine-grained recognition models. To address this, we propose an innovative approach leveraging Graph Neural Network (GNN) techniques, inspired by their recent success in various domains (Xu et al., 2019; Wu et al., 2020). By autonomously extracting fine-grained information from high-level features, our method seamlessly propagates information across object regions, offering a promising solution to the FGVC problem. Critically, it eliminates the need for extensive external annotations while significantly enhancing recognition accuracy. This transition from reliance on manual annotations to automated feature-driven learning represents a notable advancement in fine-grained object recognition.

**Fig. 2:**
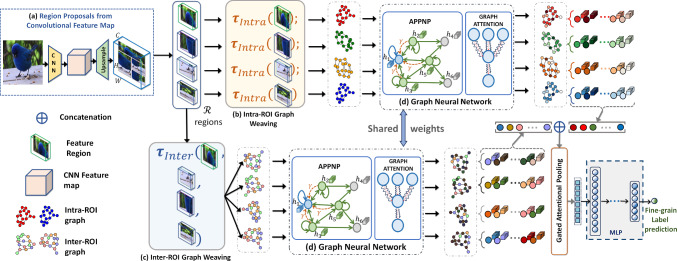
The overview of the proposed I2-HOFI framework. It consists of **a** CNN backbone for high-level pooled features from various regions of interest (ROI); **b** Intra-ROI Graph Weaving method strategically emphasizes intricate feature interactions within an ROI, aiming to capture subtle variations within specific parts of an object; **c** Inter-ROI Graph Weaving mechanism adeptly models interactions between ROIs, capturing long-range dependencies and revealing intricate global patterns; **d** Graph Neural Network (GNN) driven strategic learning mechanism by synergizing the topic-sensitive APPNP (Klicpera et al., 2019) message-passing algorithm with graph attention (Veličković et al., 2018). Feature aggregation from graph nodes is performed through gated attentional pooling before the classification

To enhance the robustness of our representation for discerning subtle variations, we introduce the inter- and intra-region high-order feature interaction mechanism. This mechanism is crafted to foster interactions across spatial and channel features extracted from a deep layer of a backbone CNN, specifically related to distinct key Regions Of Interest (ROIs) within a given object image. Our approach follows a two-stage process, as conceptually illustrated in Fig. 2. Firstly, the backbone CNN extracts high-level visual features from an input image. These features are then upsampled and pooled using geometrically constrained regions of varying sizes and positions, as depicted in Fig. 2a. Secondly, the inter- and intra-region mechanism uses GNN layers to facilitate interactions across and within the pooled regions, respectively (Fig. 2). To overcome the challenges associated with over-smoothing and a potentially extensive number of learnable parameters in GNNs, we integrate the topic-sensitive PageRank method, specifically leveraging the Approximate Personalized Propagation of Neural Predictions (APPNP) (Klicpera et al., 2019) message-passing algorithm. This choice ensures linear computational complexity and serves as the foundational layer of our designed GNN. Following this, we incorporate a Graph Attention (GAT) (Veličković et al., 2018) that uses self-attention as an attention mechanism within graph nodes. This empowers the model to dynamically learn attention weights for self-attention, fostering a more flexible exchange of information between nodes. To enhance stability and mitigate the vanishing gradient problem, we adapt these into residual connections, denoted as Residual APPNP and Residual GAT. Lastly, we introduce a novel gated attentional pooling mechanism inspired by the Gated Recurrent Unit (GRU) (Li et al., 2016) within the context of graph structures. This unique approach enables effective control of information flow within the graph, reducing the risk of overfitting by preventing the model from memorizing noise in the data. The gated attentional pooling mechanism is tailored to prioritize the most relevant representation of inter- and intra-region information exchange. Thus, the primary contribution of our work can be summarized as follows:

1. Introducing our innovative Inter and Intra Region High-Order Feature Interaction mechanism termed I2-HOFI, which autonomously extracts pertinent fine-grained object information from high-order features. This mechanism significantly amplifies interactions among object parts within a high-dimensional space, a phenomenon we term high-order feature interaction.
2. Our approach utilizes graph-based learning to propagate information both among and within object regions. It combines the topic-sensitive PageRank method, APPNP, and GAT that includes a self-attention mechanism in a novel way. Additionally, we adapt these mechanisms into residual structures (Residual APPNP and Residual GAT), to enhance stability and gradient handling.
3. Our approach alleviates the necessity for labor-intensive part-based annotations by embracing automated feature-driven learning. Notably, our method stands out for its simplicity compared to other part-annotation-free methods, all while delivering state-of-the-art performance. Rigorous experiments conducted on benchmark datasets robustly validate the effectiveness of our approach.

The remaining content is structured as follows: Sect. 2 provides an overview of related efforts in FGVC. Section 3 outlines the proposed framework. Section 4 covers the experimental outcomes, while Sect. 5 delves into a comprehensive ablation study. Finally, Sect. 6 presents the conclusion.

## Related Work

Our work is closely tied to weakly-supervised methods involving object parts, GNN approaches, and high-order feature interactions in FGVC. We provide a brief survey of these techniques in the following subsection.

### Object-Parts Based Methods

The exploration of informative relationships among object parts is paramount and has been extensively studied in Zhang et al. (2016); Yao et al. (2017); Ge et al. (2019) to achieve robust discrimination. Distinguishing subtle variations involves selecting distinct object parts across various scales, as seen in Zhang et al. (2016), where object proposals aid in this process. In Yao et al. (2017), deep features are harnessed to generate an objectness map, utilized for both part-level and object-level descriptions, and subsequently fused to enhance visual discrimination. Complementary part localization is achieved through iterative object detection and instance segmentation pipelines, as demonstrated in Ge et al. (2019). Here, a Long Short-Term Memory (LSTM) is additionally employed to encode contextual details. Similarly, local details are garnered from separate patches by dividing the entire image into smaller segments, as highlighted in Chen et al. (2019); Zhong et al. (2020); Wang et al. (2019). To refine finer details and comprehend semantic correlations within sub-regions, (Chen et al., 2019) introduces a region confusion technique that randomly disrupts the global image structure. Conversely, (Zhong et al., 2020) employs random erasing to introduce noise through part occlusion, thereby aiding in the selection of informative patches. The refinement of discriminatory patches, involving the learning of correlation weight coefficients between regions, is achieved using a region grouping sub-network, as outlined in Wang et al. (2019). Moreover, the fusion of vision and language modalities is observed in He and Peng (2017), where saliency and co-segmentation techniques are used for object localization, while cross-modal analysis is applied to correlate natural language descriptions with discriminative object parts. To identify hierarchical discriminative regions across multiple granularities and determine their optimal count for enhanced accuracy, (He et al., 2019) employs a multi-scale and multi-granularity deep reinforcement learning approach. Most of the aforementioned approaches converge on a shared focus, prioritizing the identification of informative object parts, followed by the extraction of expressive feature descriptors. In a notable departure, our approach obviates the need for explicit object part-level information. Specifically, our feature weaving techniques utilize specialized graph structures that implicitly emphasizes ROIs from a list of deformable ROIs to be more relevant to object parts, enhancing their importance while decreasing the significance of background regions. This is achieved through our message passing and graph attention mechanisms within the GNN framework, collectively enhancing the model’s discriminative power by focusing on relevant features and minimizing background noise. This innovation highlights the robustness and autonomy of our feature extraction approach.

### Bilinear Pooling-Driven Approaches

Previous researches (Lin et al., 2015; Yu et al., 2018; Hu et al., 2019) have explored various methods for integrating global and local features in FGVC through bilinear pooling mechanism. The Bilinear CNN method (Lin et al., 2015) employs bilinear pooling to capture second-order interactions between feature maps from two different CNN architecture streams, effectively integrating global and local information. While this approach is powerful, it often results in two backbones with high-dimensional feature representations, leading to increased computational complexity and potential overfitting. Unlike the Bilinear CNN method, we use graph neural networks (GNNs) to construct inter-region and intra-region graphs using high-level features from a single backbone, capturing both long-range dependencies and fine-grained local details. This graph-based representation enables efficient feature interaction without the dimensionality explosion inherent in bilinear pooling. Hierarchical Bilinear Pooling (Yu et al., 2018) extends the bilinear pooling method by introducing a hierarchical structure to model interactions at multiple levels of the deep feature hierarchy, enhancing the integration of global and local features. Although this method effectively captures multi-level feature interactions and improves the model’s ability to differentiate between fine-grained categories, it suffers from computational challenges due to several matrix multiplication operations. We differentiate ourselves by employing a shared GNN framework that utilizes the APPNP and attention mechanisms to propagate information across different regions of an image. Our method constructs inter-ROI and intra-ROI graphs to explicitly model both global patterns and local details. These graph structures naturally capture intricate feature interactions corresponding to different parts of the same object, offering a more flexible and scalable solution compared to hierarchical bilinear pooling. Furthermore, our approach avoids the high computational complexity and potential overfitting associated with bilinear pooling methods by utilizing more efficient graph-based learning. The approach described in Hu et al. (2019) enhances FGVC through weakly supervised data augmentation using bilinear attention pooling. This method combines bilinear pooling with attention mechanisms to capture and emphasize key regions, resulting in an enhanced feature representation that encapsulates both spatial and channel-wise interactions. Unlike this method, which relies on bilinear pooling and attention mechanisms, our approach leverages the structured representation and propagation capabilities of graphs. Bilinear attention pooling combines features in a linear fashion, whereas our approach facilitates high-order feature interactions using GNNs, which inherently involve non-linear transformations at each layer. This enables our model to capture complex dependencies and subtle variations within the data, leading to superior performance in FGVC tasks.

### Graph Neural Networks (GNN)

GNN has emerged as a potent tool for addressing problems involving non-Euclidean data. It excels in facilitating seamless message transmission among neighboring nodes, thus enhancing overall performance (Kipf & Welling, 2017). While its effectiveness has been demonstrated in various domains such as zero-shot recognition, multi-label image recognition, image captioning, and visual question answering (Wu et al., 2020), its potential in FGVC remains largely unexplored. Recently, Han et al. (2022) propose Vision GNN, a novel approach that treats an image as a graph of nodes, leveraging the power of GNNs for image recognition tasks. It focuses on constructing a graph representation of an image where each node corresponds to a local region, and edges represent the relationships between these regions. This approach effectively captures spatial dependencies and enhances the model’s ability to learn from complex visual structures. Chaudhuri et al. (2024) introduce Transitivity Recovering Decompositions, a method aimed at interpreting and recovering robust fine-grained relationships in visual data. Their approach decomposes complex visual relationships into simpler transitive components, making the learned representations more interpretable and robust to variations in the data. This method emphasizes the importance of understanding the fine-grained relationships within the visual content to improve classification performance. In Wang et al. (2020a), GNN is employed to extract latent attributes by modeling semantic correspondences among distinct regions within the same sub-category. Similarly, Wang et al. (2020b) leverages region correlations to identify informative regions through a criss-cross graph propagation sub-network and unified correlation feature framework. However, both methods are confined to a few regions per image (e.g., 4), potentially suboptimal for building and propagating contextual information within sub-networks crucial for effective context modeling in FGVC. Graph-based relation discovery (GaRD) (Zhao et al., 2021) learns positional and semantic feature relationships, utilizing feature grouping to address FGVC. GaRD focuses on building a contextual understanding of high-order relationships by forming a high-dimensional feature bank that extracts and stores numerous features representing different key regions of the entire image. While effective, this method can be limiting for FGVC tasks where subtle differences within localized parts of the image are crucial. In contrast, we introduce a unified GNN-based approach that learns to simultaneously propagate information within and across multiple pooled deformable ROIs. This is achieved through a thoughtful combination of the topic-sensitive PageRank approximation (Klicpera et al., 2019) and Graph Attention (GAT) (Veličković et al., 2018). This design enables the capture of finer details by facilitating information exchange not only between colocated positions of different regions but also among their neighboring regions. When compared to other graph-based approaches like GCL (Wang et al., 2020b), which focuses on learning region correlations through Criss-cross Graph Propagation (CGP) and enhancing features using Correlation Feature Strengthening (CFS), our method introduces a uniquely designed intra- and inter-ROI graph structure that explicitly dictates the exchange and highlighting of relevant information via an attention mechanism. This design not only provides more effective interactions among features but also leverages high-order feature interactions both within and between regions, a capability that GCL lacks. Additionally, we incorporate residual connections (Residual APPNP and Residual GAT) to enhance stability and gradient handling, addressing common issues like over-smoothing and extensive parameter learning typically found in their non-residual GNN counterparts.

### High-Order Feature Interactions

Feature learning is pivotal in advancing computer vision tasks such as image retrieval, semantic segmentation, object detection, and tracking. The success of CNNs stems from their adeptness at discerning discriminative features directly from raw pixel data. Initially, fully connected layer activations served as prevalent image representations; however, the realization that deeper convolutional layers harbor valuable mid and high-level information, including object parts and complete objects (Zeiler et al., 2011), spurred the widespread adoption of convolutional features and descriptors in computer vision tasks. The application of encoding techniques to local convolutional descriptors has significantly enhanced performance compared to using fully connected layer outputs, partly due to the inclusion of higher-order statistics in the final features. Notably, the covariance matrix-based representation (Wang et al., 2015, 2020) has been instrumental in capturing second-order feature interactions, finding applications in both computer vision and machine learning. The integration of this representation with deep descriptors (Song et al., 2022) has yielded methods showcasing promising accuracy in fine-grained recognition tasks. A representative example is Bilinear CNNs (Lin et al., 2015), which models images as the pooled outer product of features extracted from two deep CNNs, effectively encoding second-order statistics and leading to substantial improvements in fine-grained recognition. However, the outer product operation introduces high dimensional features, posing challenges like overfitting, particularly in large-scale applications. Addressing the dimensionality challenge, recent research has explored techniques such as Tensor Sketch (Pham & Pagh, 2013) and low-rank approximation (Kong & Fowlkes, 2017), aiming to approximate the second-order statistics of the original bilinear pooling operation while concurrently reducing feature dimensions. Additionally, strategies like quadratic transformation with a low-rank constraint (Li et al., 2017) and dimension reduction projections (Yu et al., 2018) have been proposed to alleviate the issue of dimension explosion. Certain approaches aim to model higher-order interactions beyond the second order, crafting more potent and discriminative feature representations. Techniques such as kernel pooling (Cui et al., 2017), polynomial kernel-based predictors (Cai et al., 2017), and DeepKSPD (Engin et al., 2018) jointly learn deep local descriptors and kernel-matrix-based covariance representations in an end-to-end trainable manner. To enhance discriminative features, *L*2 feature normalization, a prevailing practice (Wei et al., 2018; Sánchez et al., 2013), is applied to mitigate common patterns of high response. However, it is essential to note that the sole use of *L*2-normalization can introduce instability in high-order information and hinder convergence. To overcome these challenges, researchers explore nonlinear scaling based on Singular Value Decomposition (SVD) or eigendecomposition (EIG) to enhance the stability of second-order representations (Li et al., 2017; Wang et al., 2017). Graph-based models (Zhao et al., 2021; Xie et al., 2023) offer innovative ways to efficiently model high-level feature interactions. Graphs, adept at capturing complex relationships and dependencies between features, construct a structured representation of feature interactions. Nodes represent individual features or feature groups, and edges denote interactions or relationships. Applying graph convolution propagates information across nodes, facilitating principled and efficient feature interactions. This captures high-level feature interactions while inherently encoding feature relationships for interpretability. Effective techniques like subgraph sampling (Hübler et al., 2008) and hierarchical graph representations manage computational complexity, enabling the analysis of high-order interactions across diverse domains, from FGVC to scene understanding.

In summary, high-level feature interactions have notably enhanced computer vision tasks, particularly FGVC. Current advancements in this field are significant, with the integration of graph-based models showing promising potential for further improving the efficiency and effectiveness of feature interaction modeling.

## Proposed Approach

The proposed I2-HOFI architecture is shown in Fig. 2. It processes an input image, extracting a high-level convolutional feature map using multiple overlapping deformable ROIs. A high-order interaction graph is constructed, connecting different segments of the deep feature map of the CNN both within (intra) and across (inter) the ROIs. The proposed residual GNN with APPNP and GAT are subsequently explored to facilitate the relevant information flow within and between these regions, enhancing information-rich discriminative features. These features are then pooled to create a discriminative feature embedding, forming a robust foundation for advancing FGVC.

### Problem Formulation

In the context of training an image classifier, a dataset consists of $N$ images denoted as ${\mathcal {I}}=\{I_n|n=1, 2, \dots , N\}$, accompanied by their corresponding class labels $y_n$. The main objective is to guide the classifier in learning a mapping function ${\mathcal {F}}$, which generates predictions ${\hat{y}}_n={\mathcal {F}}\left( I_n\right)$ to match the true label $y_n$ closely. During the training process, the function ${\mathcal {F}}$ is continually refined by minimizing a loss function denoted as ${\mathcal {L}}\left( y_n,{\hat{y}}_n\right)$, which quantifies the dissimilarity between the predicted and actual labels. Here, ${\mathcal {F}}$ represents an end-to-end deep neural network. To advance FGVC, our sophisticated intra- and inter-region high-order feature interaction framework meticulously captures intricate relationships among high-order features derived from a CNN backbone. The process initiates with images being processed through the CNN backbone, resulting in the extraction of upsampled CNN features. The generation of proposal deformable regions, characterized by varying dimensions, is accomplished by thoughtful consideration of feature segments across spatial and channel dimensions. This strategic approach leads to the formation of two distinct sets of interaction graphs: intra-region graphs that establish connections among segments within a region, and inter-region graphs that link segments across co-located positions. Leveraging our attention-driven message-passing mechanism, we skillfully combine relevant information both within and across regions. This deliberate emphasis on the most pertinent feature segments significantly amplifies discriminative power. As a final refinement, a sophisticated pooling mechanism aggregates the refined information, providing an additional boost to the overall discriminative power of the features. In essence, our comprehensive framework not only enhances our understanding of intricate visual details but also substantially contributes to the precision and discriminatory capabilities of our framework.

### CNN Features and Deformable ROIs

**Fig. 3:**
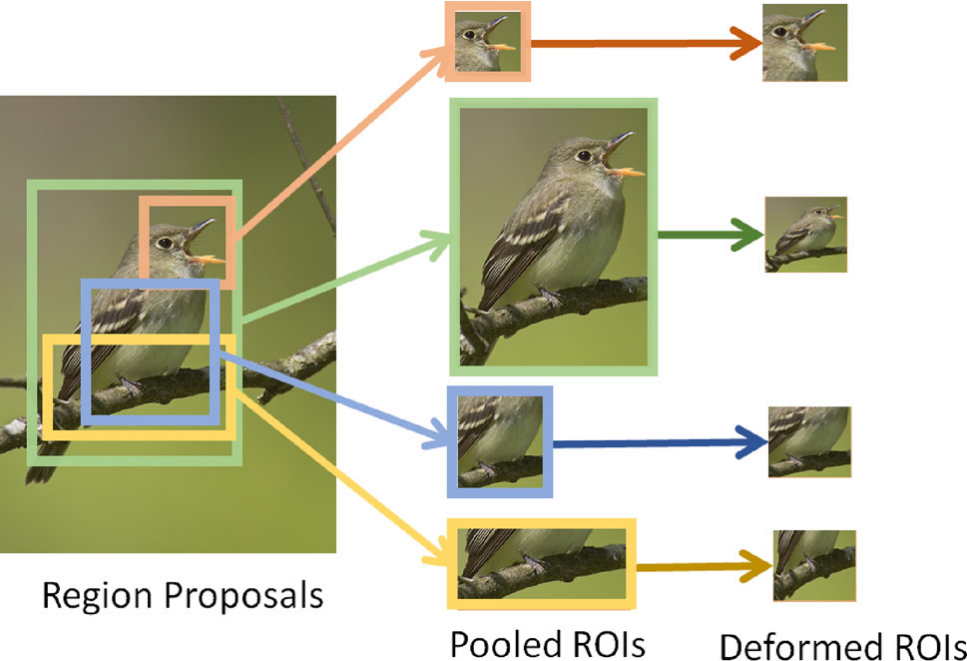
Selected ROIs with varying dimensions from the upscaled CNN feature map (shown over the original image), processed using bilinear pooling (4 examples shown for clarity)

We use the lightweight Xception backbone (Chollet, 2017) to extract and upsample CNN features, following the methodology outlined in CAP (Behera et al., 2021). For the generation of ROIs, we implement a modified version of deformable ROI pooling (Dai et al., 2017), deviating from the standard approach of learning offsets for each grid cell. Instead, we integrate the computation of the Histogram of Oriented Gradients (HOG) (Behera et al., 2020), to examine cells and blocks more closely. Given the input convolutional feature map *f* (width *w*, height *h*, and channel *C*), ROI pooling divides this into $$\kappa \times \kappa $$ spatial cells like in HOG computation. In deformable pooling, the spatial offsets {$$\Delta $$ $$p_{i,j}|$$ $$0\le i,j<\kappa $$} are added to the position (top-left corner), width and height of an ROI to generate another one. For example, if we start with the top-left cell ($$i=0, j=0)$$ in *f* as an ROI then we can create a set of ROIs by applying this offset to position, or width or height, and their combinations. This approach generates variable-sized regions, transforming them into fixed-dimension ROIs with the capacity to deform and fit diverse object shapes (see Fig. 3). The image $$I_n$$ is now represented by $${\mathcal {R}}$$ ROIs. These ROIs are broken down into multiple non-overlapping segments, denoted by the set $${\mathcal {N}}$$. This segmentation is achieved by dividing the original feature map along the channel dimension *C* at each spatial point, creating a collection of non-overlapping feature segments $$\{f_1, f_2, \ldots , f_n\}$$, where $$n = |{\mathcal {N}}|$$ signifies the total number of segments. Mathematically, this partitioning is expressed as:

1 $$\begin{aligned} f_{roi} = \bigcup _{s=1}^{n} f_s \end{aligned}$$

where, $f_{roi}\in {\mathcal {R}}$ represents an ROI feature map with dimension ${\mathbb {R}}^{W \times H \times C}$ with *W*, *H* and *C* representing width, height and channel dimension respectively, while $f_s$ represents the $s^{th}$ non-overlapping segment with dimensions ${\mathbb {R}}^{1 \times 1 \times C'}$. Here, $C' = C / 2^k$ delineates the partitioned channel dimension, with *k* being a positive integer. Thus, we have a total of $n = W \cdot H \cdot 2^k$ distinct feature segments within the ROI. The union operator $\cup$, symbolizes the recombination of all individual segments $f_s$ into the original ROI feature map $f_{roi}$.

**Fig. 4:**
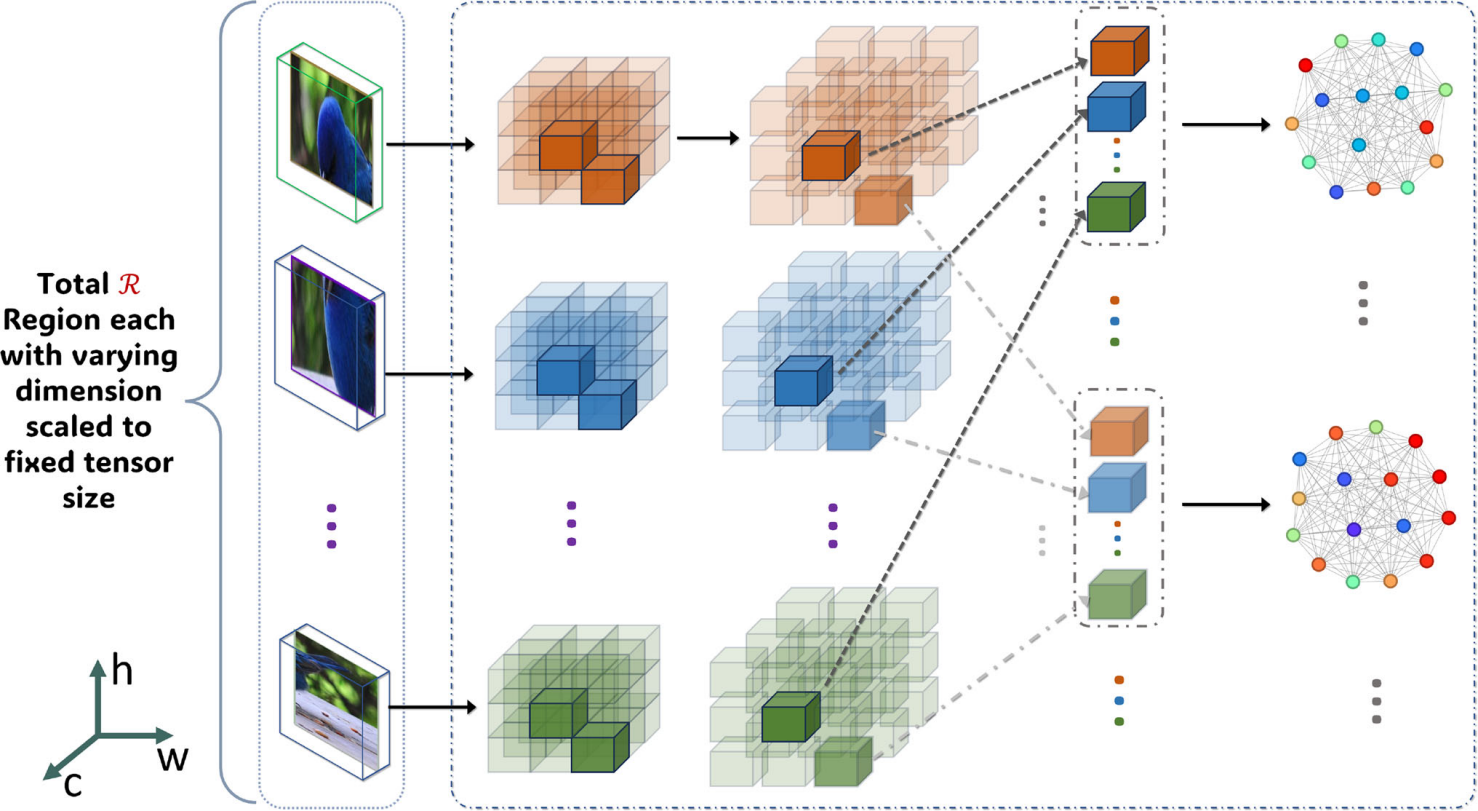
The Inter-ROI interaction mechanism is formed by building graphs between distinct feature segments of ROIs from corresponding co-located positions

### Inter-ROI Graph Weaving

This section provides details on how we form the inter-ROI feature interaction mechanism by constructing a series of graphs across the ${\mathcal {R}}$ ROIs. Each ROI consists of *n* segments. We choose one segment from each co-located position $f_s$ based on the partitioning of $f_{roi}$, as depicted in Fig. 4. This results in the creation of *n* graphs, with each graph corresponding to one of these *n* segments with ${\mathcal {R}}$ nodes. Each segment is a 1D feature vector of length $C'$. These vectors serve as the node features in our graphs. We designate each inter-ROI graph as $G_{inter} = ({\mathcal {R}}, {\mathcal {E}}_{inter})$, where ${\mathcal {E}}_{inter}$ represents edges linking ${\mathcal {R}}$ nodes. $G_{inter}$ is a fully connected graph without self-loop, leading to $|{\mathcal {E}}_{inter}| = {|{\mathcal {R}}| \cdot (|{\mathcal {R}}| - 1)} / {2}$ edges and adjacency matrix ${\textbf{A}}_{inter} \in {\mathbb {R}}^{|{\mathcal {R}}| \times |{\mathcal {R}}|}$. Hence, the matrix ${\textbf{A}}_{inter}$ efficiently encapsulates extensive interconnections among the ROIs, establishing a robust framework for analyzing feature interactions. This interaction mechanism facilitates collaboration among diverse high-level features, promoting the exchange of contextual information across different parts of an object. Unlike traditional convolution filters, this method adeptly captures long-range dependencies, unveiling global patterns and facilitating information sharing among co-located feature segments. As a result, it enhances the discriminability by incorporating global context and dependencies.

### Intra-ROI Graph Weaving

**Fig. 5:**
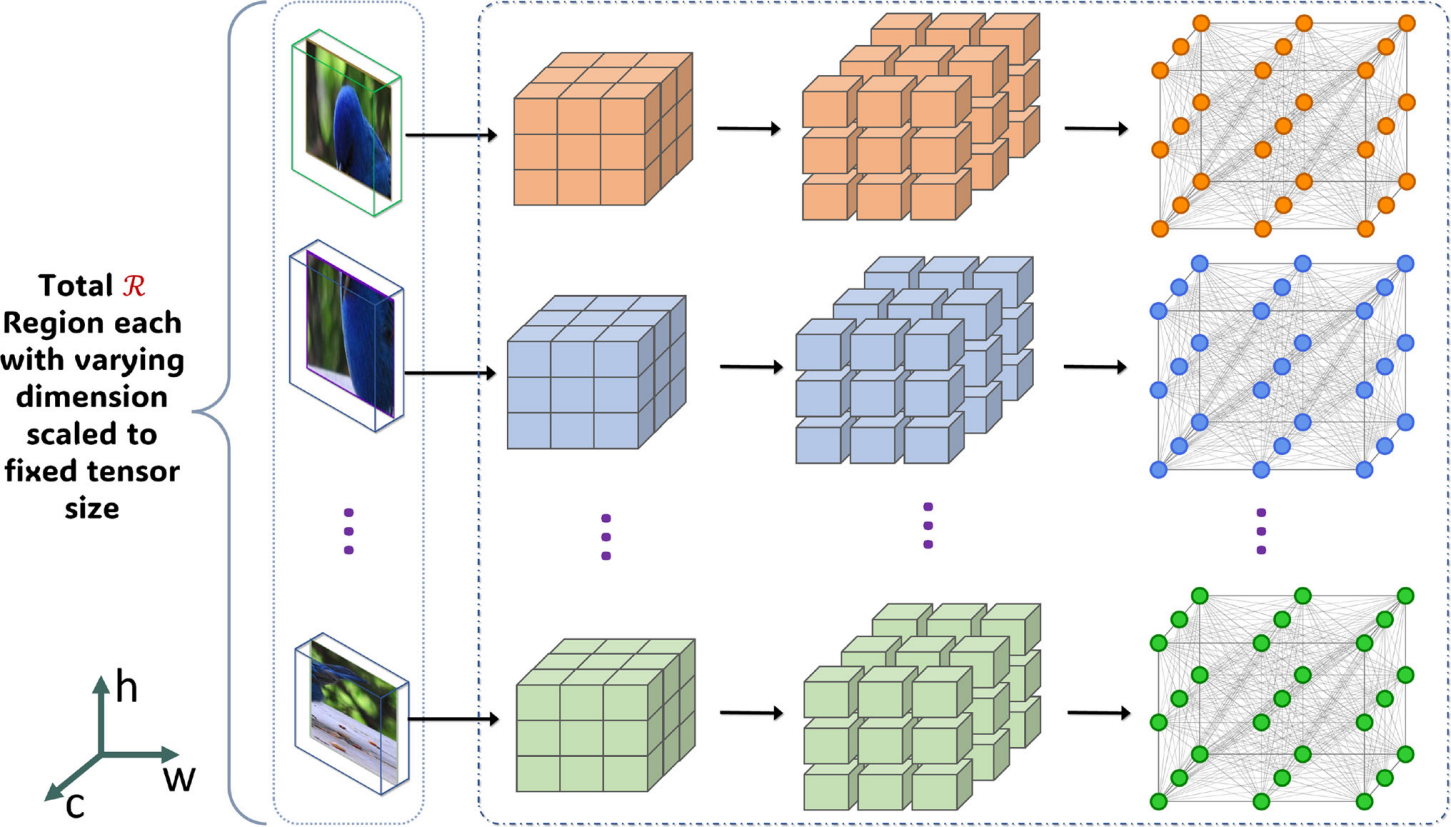
Intra-feature weaving mechanism through graphs constructed among tensor segments of high-level representation of image regions

In parallel to the inter-ROI graph $G_{inter}$, the intra-ROI graph $G_{intra}$ captures the feature interaction by facilitating the flow of information within the *n* distinct segments of each ROI independently. In this context, ‘independently’ denotes the restriction of information flow exclusively within each ROI, ensuring a focused and localized analysis of features. Similar to the inter-ROI graph weaving mechanism, the intra-ROI configuration is constructed over the identical set of *n* segments. However, the approach diverges as a graph is delineated for the *n* segments within each of the ${\mathcal {R}}$ Regions individually. Consequently, each ROI is represented by a separate graph, encompassing *n* nodes (${\mathcal {R}}$ nodes in inter-ROI graph). This leads to the creation of ${\mathcal {R}}$ distinct yet structurally analogous graphs (*n* graphs in inter-ROI) and is represented as $G_{intra} = ({\mathcal {N}}, {\mathcal {E}}_{intra})$, with ${\mathcal {E}}_{intra}$ edges interlinking the *n* nodes without self-loop. $G_{intra}$ is fully-connected (Fig. 5), resulting in $|{\mathcal {E}}_{intra}| = \frac{n \cdot (n - 1)}{2}$ edges represented with an adjacency matrix ${\textbf{A}}_{intra} \in {\mathbb {R}}^{n \times n}$. The $G_{intra}$ graph adeptly encapsulates the interconnections among feature segments within an ROI, fostering intricate feature interactions within a specific region. This also serves as a critical tool in understanding and quantifying the intra-ROI feature interactions via a localized graph-based approach. This mechanism encourages collaboration among similar high-level features, fostering an exchange of information to capture subtle variations within a part of an object. Consequently, it elevates discriminability by incorporating local context and dependencies, thereby contributing to the richness and depth of the analytical process.

### Learning Interactions Using Residual GNN

Once the $G_{inter}$ (Sect. 3.3) and $G_{intra}$ (Sect. 3.4) graphs are constructed, the goal is to capture the intricate relationships between regions/nodes and graphs, thereby enhancing the feature representation of the entire image and making it more discriminative. To accomplish this, we apply the GNN principle to process the graphs $G_{inter}$ and $G_{intra}$ through a shared learning mechanism. This shared mechanism is feasible due to the uniform node feature dimension ($C'$) across all graphs, as outlined in Sect. 3.2. The input feature set for each $G_{inter}$ graph (Fig. 4) defined as ${\textbf{X}}_{inter, i} = \{f_{ij}\}^{|{\mathcal {R}}|}_{j=1}$ with the corresponding transformed output feature set is ${\textbf{Y}}_{inter, i} = \{{\hat{f}}_{ij}\}^{|{\mathcal {R}}|}_{j=1}$, where, the index $i=1,\dots ,n$ runs through the disjoint segments of each $f_{roi}$. Conversely, for $G_{intra}$ (Fig. 5), the input feature set as ${\textbf{X}}_{intra, j} = \{f_{ij}\}^{n}_{i=1}$ and its transformed output as ${\textbf{Y}}_{intra, j} = \{{\hat{f}}_{ij}\}^{n}_{i=1}$, with the index $j=1,\dots ,|{\mathcal {R}}|$ iterating over each region. Therefore, our model processes $n + |{\mathcal {R}}|$ distinct graphs, addressing inter- and intra-ROI interactions. The goal is to represent the $n + |{\mathcal {R}}|$ graphs with a single adjacency matrix ${\textbf{A}}$ by combining all ${\textbf{A}}_{inter} \in {\mathbb {R}}^{|{\mathcal {R}}| \times |{\mathcal {R}}|}$ and ${\textbf{A}}_{intra} \in {\mathbb {R}}^{n \times n}$ adjacency matrices of the respective $G_{inter}$ and $G_{intra}$ graphs. To achieve this, we arrange the individual adjacency matrices ${\textbf{A}}_{inter,i}$ along the diagonal of the larger matrix ${\textbf{A}}_{inter}$. The non-diagonal portions of ${\textbf{A}}_{inter}$ are filled with zero matrices ${\textbf{0}}_{|{\mathcal {R}}|} \in {\mathbb {R}}^{|{\mathcal {R}}| \times |{\mathcal {R}}|}$, signifying no connections between nodes of different subgraphs. The resultant matrix ${\textbf{A}}_{inter}$ is expressed as:

2 $$\begin{aligned} {\textbf{A}}_{inter} = \begin{bmatrix} {\textbf{A}}_{inter,1} & {\textbf{0}}_{|{\mathcal {R}}|} & \cdots & {\textbf{0}}_{|{\mathcal {R}}|} \\ {\textbf{0}}_{|{\mathcal {R}}|} & {\textbf{A}}_{inter,2} & \cdots & {\textbf{0}}_{|{\mathcal {R}}|} \\ \vdots & \vdots & \ddots & \vdots \\ {\textbf{0}}_{|{\mathcal {R}}|} & {\textbf{0}}_{|{\mathcal {R}}|} & \cdots & {\textbf{A}}_{inter,n} \end{bmatrix} \end{aligned}$$

The above matrix ${\textbf{A}}_{inter} \in {\mathbb {R}}^{(n|{\mathcal {R}}|) \times (n|{\mathcal {R}}|)}$ represents the total $n \times |{\mathcal {R}}|$ nodes. Similarly, the adjacency matrix ${\textbf{A}}_{intra}$ for all $G_{intra}$ graphs (refer to Sect. 3.4) is structured by swapping $|{\mathcal {R}}|$ and *n* in Eq. (2). This is represented as:

3 $$\begin{aligned} {\textbf{A}}_{intra} = \begin{bmatrix} {\textbf{A}}_{intra,1} & {\textbf{0}}_{n} & \cdots & {\textbf{0}}_{n} \\ {\textbf{0}}_{n} & {\textbf{A}}_{intra,2} & \cdots & {\textbf{0}}_{n} \\ \vdots & \vdots & \ddots & \vdots \\ {\textbf{0}}_{n} & {\textbf{0}}_{n} & \cdots & {\textbf{A}}_{intra,|{\mathcal {R}}|} \end{bmatrix} \end{aligned}$$

To facilitate GNN-driven message passing and aggregation mechanisms, we combine the above ${\textbf{A}}_{inter}$ and ${\textbf{A}}_{intra}$ adjacency matrices into a single matrix ${\textbf{A}}$:

4 $$\begin{aligned} {\textbf{A}} = \begin{bmatrix} {\textbf{A}}_{inter} & {\textbf{0}}_{n|{\mathcal {R}}|} \\ {\textbf{0}}_{n|{\mathcal {R}}|} & {\textbf{A}}_{inter} \end{bmatrix} \end{aligned}$$

The zero matrix ${\textbf{0}}_{n|{\mathcal {R}}|} \in {\mathbb {R}}^{(n|{\mathcal {R}}|) \times (n|{\mathcal {R}}|)}$ ensures that there are no connections between the inter and intra graphs. This structure enables the processing of both sets of graphs within a shared GNN framework while preserving their distinct characteristics.

The final feature set is represented by the union of all features from both sets of graphs: ${\textbf{X}} = \{{\textbf{X}}_{inter,i}\}^{n}_{i=1} \bigcup \{{\textbf{X}}_{intra,j}\}^{|{\mathcal {R}}|}_{j=1}$, and the corresponding transformed output feature set is denoted as ${\textbf{Y}} = \{{\textbf{Y}}_{inter,i}\}^{n}_{i=1} \bigcup \{{\textbf{Y}}_{intra,j}\}^{|{\mathcal {R}}|}_{j=1}$. In practice, the transformed features of inter and intra graphs are often concatenated, yielding a comprehensive matrix ${\textbf{Y}}$. We streamline the representation of the combined inter and intra graphs into a single graph, denoted as *G* over the total vertices $n \times {\mathcal {R}}$. This is concisely expressed as follows:

5 $$\begin{aligned} G = \left( \bigcup _{i=1}^{n} G_{inter,i} \right) \bigcup \left( \bigcup _{j=1}^{|{\mathcal {R}}|} G_{intra,j} \right) \end{aligned}$$

The final adjacency matrix ${\textbf{A}}$, with an added self-loop, is denoted as ${\hat{\textbf{A}}} = {\textbf{A}} + {\textbf{I}}_{n|{\mathcal {R}}|}$, where ${\textbf{I}}_{n|{\mathcal {R}}|} \in {\mathbb {R}}^{n \times |{\mathcal {R}}|}$ is the identity matrix. The normalized adjacency matrix used in the GNN is ${\mathcal {A}} = {\hat{\textbf{D}}}^{-1/2} {\hat{\textbf{A}}} {\hat{\textbf{D}}}^{-1/2}$, with ${\hat{\textbf{D}}}$ being the diagonal node degree matrix of ${\hat{\textbf{A}}}$. In general, a typical message passing algorithm GNN (Kipf & Welling, 2017) utilizes a layer-wise propagation rule: ${\textbf{X}}^{(l)} = \sigma ({\mathcal {A}} {\textbf{X}}^{(l-1)}{\textbf{W}}^{(l-1)})$, where ${\textbf{X}}^{(0)}={\textbf{X}}$, ${\textbf{Y}}={\textbf{X}}^{(L)}$, and *L* denotes the number of layers. Here, ${\textbf{W}}^{(l)}$ is the weight matrix for the *l*-th layer, and $\sigma (\cdot )$ is a non-linear activation function (e.g., ReLU). Traditional GNNs often utilize an averaging aggregation, which, when employing too many layers, can lead to over-smoothing and a diminished focus on the local neighborhood. In GNNs, a larger neighborhood typically increases the depth and the number of learnable parameters, as common aggregation schemes involve learnable weight matrices in each layer. To address these challenges, we incorporate the approximate personalized propagation of neural predictions (APPNP) message-passing algorithm (Klicpera et al., 2019) into our residual GNN. This adaptation includes a skip connection, contributing to the model’s robustness. The APPNP approach achieves linear computational complexity by approximating a topic-sensitive PageRank algorithm through power iteration, akin to a random walk with restarts. The power iteration step is formulated as:

6 $$\begin{aligned} \begin{aligned} {\textbf{H}}^{(0)}&= \text {MLP}({\textbf{X}};\theta ), \\ {\textbf{H}}^{(p)}&= (1-\gamma ){\mathcal {A}}{\textbf{H}}^{(p-1)} + \gamma {\textbf{H}}^{(0)},\\ {\textbf{Y}}&= {\textbf{H}}^{(p)} + {\textbf{X}} \end{aligned} \end{aligned}$$

In this formulation, $\gamma \in (0,1]$ represents the teleport probability, signifying the likelihood of the propagation process ‘restarting’ at a root node in each step, where *p* is the number of power iteration steps. In this context, MLP, a multi-layer perceptron with parameter $\theta$, predicts ${\textbf{H}}$, preserving the local neighborhood characteristics and serving as both the starting vector and teleport set. Finally, we add the initial ${\textbf{X}}$ to ${\textbf{H}}^{(p)}$ to establish the residual (skip) connection. It is worth noting that MLP operates on each node’s feature independently, enabling parallelization. Advancing to the next stage in our GNN, we introduce a residual graph attention mechanism to calculate the hidden representations of each node in the graph *G*. This involves attending over its neighbors, employing the self-attention strategy in Veličković et al. (2018). Each node ${\textbf{z}}_{u \in n|{\mathcal {R}}|} \in {\textbf{Y}}$ is updated as follows:

7 $$\begin{aligned} \begin{aligned} {\textbf{z}}_u'&= \sigma \Biggl ( \alpha _{uu}^{(l-1)} {\textbf{z}}_u^{(l-1)} {\textbf{W}}^{(l-1)}_1 \Biggr .\\ \Biggl .&\quad + \sum _{v \in N(u)} \alpha _{uv}^{(l-1)} {\textbf{z}}_v^{(l-1)} {\textbf{W}}^{(l-1)}_1 + {\textbf{b}}^{(l-1)}_1 \Biggr ) \end{aligned} \end{aligned}$$

In this formulation, ${\textbf{z}}_u'$ represents the updated embedding for node *u* at layer $l-1$. The function $\sigma$ is a non-linear activation function, such as ReLU. *N*(*u*) denotes the neighborhood of node *u*, and $\alpha _{uv}^{(l-1)}$ are the attention coefficients that determine the importance of node *v* to node *u* at layer $l-1$. The attention coefficients are computed as follows:

8 $$\begin{aligned} \alpha _{uv} = \frac{\exp \left( \text {LeakyReLU}\left( {\textbf{a}}^{T} \left[ {\textbf{z}}_u{\textbf{W}}_1 \Vert {\textbf{z}}_v {\textbf{W}}_1 \right] \right) \right) }{\sum _{q \in N(u) \cup \{u\}} \exp \left( \text {LeakyReLU}\left( {\textbf{a}}^{T} \left[ {\textbf{z}}_u{\textbf{W}}_1 \Vert {\textbf{z}}_q {\textbf{W}}_1 \right] \right) \right) } \nonumber \\ \end{aligned}$$

where $\Vert$ is the concatenation operation; ${\textbf{a}} \in {\mathbb {R}}^{2F'}$ is a trainable attention kernel with output feature dimension $F'$ and LeakyReLU activation. $\textbf{W}_1$ is a learnable weight parameter. The updated node embedding is then calculated by adding a residual (skip) connection as shown:

9 $$\begin{aligned} {\textbf{z}}_u^{(l)} = {\textbf{z}}_u' + {\textbf{z}}_u^{(l-1)} \end{aligned}$$

This approach ensures that the transformed node features for both inter and intra regions, denoted by ${\textbf{Y}}$, encapsulate high-order feature segment representations within the comprehensive constructed graph *G* in Eq. (5). The concluding step involves aggregating all node features across regions (nodes) into a singular image-level descriptor $f_t$. This aggregation is accomplished through gated attentional pooling (Li et al., 2016), as follows:

10 $$\begin{aligned} f_t = \sum _{u=1}^{n \times |{\mathcal {R}}|} \sigma (z_u^{(l)} {\textbf{W}}_2+{\textbf{b}}_2) \odot (z_u^{(l)} {\textbf{W}}_3+{\textbf{b}}_3), \end{aligned}$$

In this equation, ${\textbf{W}}_2$, ${\textbf{W}}_3$, ${\textbf{b}}_2$, and ${\textbf{b}}_3$ represent learnable parameters. The function $\sigma (\cdot )$ operates as an element-wise sigmoid, offering soft attention to determine which feature segments contribute most effectively to a discriminative representation. The Hadamard product is denoted by $\odot$. The complete set of learnable parameters in our model, as outlined in Eqs. 6, 7, and 10, is collectively denoted as $\theta _t$. This set includes $\theta$, the weight matrices ${\textbf{W}}_1$, ${\textbf{W}}_2$, ${\textbf{W}}_3$, and the bias terms ${\textbf{b}}_1$, ${\textbf{b}}_2$, ${\textbf{b}}_3$. Please note that the edges in both inter-ROI and intra-ROI graphs have scalar weights which are learnable rather than static, determined by Eq. 8 from the GAT self-attention mechanism. These weights are dynamically updated during training, enabling the model to prioritize relevant connections (higher weights) and suppress less important ones (lower weights).

## Experimental Results and Discussions

First, we introduce the datasets and outline experimental specifics. Subsequently, we compare our results to the state-of-the-art (SotA). Following this, we delve into an examination of our model’s intricacy and perform a qualitative analysis to gain deeper insights into its decision-making processes. Lastly, we conduct ablation studies to assess the contributions and effects of its critical components and parameters.

**Table 1:** Statistics of the datasets used in our experiments

Dataset	#Train / #Test	#Class	SotA	I2-HOFI
Aircraft	6667/3333	100	95.40 (Bera et al., 2022)	**96**.**42**
CUB-200	5994/5794	200	**92**.**90** (Chen et al., 2024)	91.60
Cars	8144/8041	196	96.30 (Chaudhuri et al., 2022)	**96**.**92**
Flowers	2040/6149	102	97.90 (Bera et al., 2022)	**99**.**00**
NABirds	23,929/24,633	555	91.08 (Xu et al., 2024)	**92**.**12**

Accuracy (%) of the best SotA and our I2-HOFI

### Datasets

Our approach abstains from relying on object or part bounding box annotations when evaluating its performance across five benchmark datasets, each distinguished by unique characteristics, as outlined in the Table 1: Aircraft (Maji et al., 2013), Caltech-UCSD Birds (CUB-200) (Wah et al., 2011), Stanford Cars (Krause et al., 2013), Oxford Flowers (Nilsback & Zisserman, 2008) and NABirds (Van Horn et al., 2015). To assess our model’s performance, we use top-1 accuracy (%) as the evaluation metric.

### Implementation Details

Our framework is developed using TensorFlow version 2.10.0. Following the CAP framework (Behera et al., 2021), we upsampled the CNN feature of size $7 \times 7 \times 2048$ from Xception backbone to $42 \times 42 \times 2048$. The region proposals mechanism is adapted from deformable ROI pooling in Dai et al. (2017) and computation of HOG using cells and blocks in Behera et al. (2020) to generate 27 optimal regions ($|{\mathcal {R}}|$) with varying sizes. The spatial dimension of the pooled features from each region is set to a fixed size of $3\times 3$. The output feature dimension of each node ${\textbf{z}}_{u \in n|{\mathcal {R}}|} \in {\textbf{Y}}$ is set to 512, a configuration determined to be optimal for our task. In the APPNP step, we incorporate a single-layer Multi-Layer Perceptron (MLP) with a teleport probability parameter $\gamma$ set to 0.3. The dimension of the final pooled feature $f_t$ in Eq. (10) is set to 1024.

**Table 2:** Comparison of different SotA methods on Aircraft (Maji et al., 2013) dataset

Method	Publication venue	Backbone	Accuracy (%)
GCL (Wang et al., 2020b)	AAAI’20	ResNet-50	93.20
APIN (Zhuang et al., 2020)	AAAI’20	DenseNet-161	93.90
CAMF$^{\S }$ (Miao et al., 2021)	SPL’21	SwinT-B	93.30
PMG (Chang et al., 2021)	CVPR’21	ResNet-50	93.60
SCAP (Liu et al., 2021)	TMM’21	ResNet-50	93.60
CSC (Wang et al., 2020a)	ACM MM’21	ResNet-50	93.80
APCN (Ding et al., 2021)	TIP’21	ResNet-50	94.10
GaRD (Zhao et al., 2021)	CVPR’21	ResNet-50	94.30
CAP (Behera et al., 2021)	AAAI’21	ResNet-50	94.90
SRGNN (Bera et al., 2022)	TIP’22	Xception	95.40
FRe (Zhao et al., 2023)	NN’23	DenseNet-161	94.20
SRGN$^{\S }$ (Wang et al., 2023)	IJCV’23	MetaFormer-I	94.40
GDSMP (Ke et al., 2023)	PR’23	ResNet-50	94.40
RP (Chaudhuri et al., 2022)	NeurIPS’22	ResNet-50	95.25
FET$^{\S }$ (Chen et al., 2024)	PR’24	SwinT-B	95.30
Base CNN		Xception	79.50
I2-HOFI (Ours)		ResNet-50	92.26
I2-HOFI (Ours)		Xception	**96**.**42**

$^{\S }$Denotes transformer-based approaches

**Table 3:** Comparison of different SotA methods on CUB-200 (Wah et al., 2011) dataset

Method	Publication venue	Backbone	Accuracy (%)
BARM (Liu et al., 2019)	TMM’19	DenseNet-161	89.50
CPM$^{*}$ (Ge et al., 2019)	CVPR’19	GoogleNet	90.40
APIN (Zhuang et al., 2020)	AAAI’20	DenseNet-161	90.00
GaRD (Zhao et al., 2021)	CVPR’21	ResNet-50	89.60
PMG (Chang et al., 2021)	CVPR’21	ResNet-50	89.90
CAMF$^{\S }$ (Miao et al., 2021)	SPL’21	SwinT-B	91.20
CAP (Behera et al., 2021)	AAAI’21	Xception	91.80
SRGNN (Bera et al., 2022)	TIP’22	Xception	91.90
TrnFG$^{\S }$ (He et al., 2021)	AAAI’22	ViT-B	91.70
FRe (Zhao et al., 2023)	NN’23	DenseNet-161	89.90
GDSMP (Ke et al., 2023)	PR’23	ResNet-50	89.90
RP (Chaudhuri et al., 2022)	NeurIPS’22	ResNet-50	92.00
SRGN$^{\S }$ (Wang et al., 2023)	IJCV’23	MetaFormer-I	92.50
MGFF$^{\S }$ (Xu et al., 2024)	PR’24	SwinT-B	92.66
FET$^{\S }$ (Chen et al., 2024)	PR’24	SwinT-B	**92**.**90**
Base CNN		Xception	75.60
I2-HOFI (Ours)		ResNet-50	90.12
I2-HOFI (Ours)		Xception	91.60

$^{\S }$Denotes transformer-based approaches

$^{*}$Signifies joint learning from multiple datasets to enhance accuracy

**Table 4:** Comparison of different SotA methods on Cars (Krause et al., 2013) dataset

Method	Publication venue	Backbone	Accuracy (%)
S3Ns (Ding et al., 2019)	ICCV’19	Resnet-50	94.70
GaRD (Zhao et al., 2021)	CVPR’21	Resnet-50	95.10
PMG (Chang et al., 2021)	CVPR’21	Resnet-50	95.10
APIN (Zhuang et al., 2020)	AAAI’21	DenseNet-161	95.30
CAMF$^{\S }$ (Miao et al., 2021)	SPL’21	SwinT-B	95.30
APCN (Ding et al., 2021)	TIP’21	Resnet-50	95.40
CAP (Behera et al., 2021)	AAAI’21	Xception	95.70
TrnFG$^{\S }$ (He et al., 2021)	AAAI’22	ViT-B	94.80
CSC (Wang et al., 2020a)	ACM MM.’22	Resnet-50	94.90
SRGNN (Bera et al., 2022)	TIP’22	Xception	96.10
FRe (Zhao et al., 2023)	NN’23	DenseNet-161	95.10
GDSMP (Ke et al., 2023)	PR’23	Resnet-50	95.30
SRGN (Wang et al., 2023)	IJCV’23	MetaFormer-I	96.10
RP (Chaudhuri et al., 2022)	NeurIPS’22	Resnet-50	96.30
MGFF$^{\S }$ (Xu et al., 2024)	PR’24	SwinT-B	93.33
FET$^{\S }$ (Chen et al., 2024)	PR’24	SwinT-B	95.90
Base CNN		Xception	84.80
I2-HOFI (Ours)		ResNet-50	94.33
I2-HOFI (Ours)		Xception	**96**.**92**

$^{\S }$Denotes transformer-based approaches

**Table 5:** Comparison of different SotA methods on Flowers101 (Nilsback & Zisserman, 2008) dataset

Method	Publication venue	Backbone	Accuracy (%)
PBC (Huang et al., 2016)	TMM’16	GoogleNet	96.10
IntAct (Xie et al., 2016)	CVPR’16	VGG19	96.40
SJFT$^{*}$ (Ge & Yu, 2017)	CVPR’17	ResNet-152	97.00
OPAM$^{*}$ (Peng et al., 2018)	TIP’18	VGGNet	97.10
DSTL$^{*}$ (Cui et al., 2018)	CVPR’18	Inception-v3	97.60
Cos.Ls$^{*}$ (Barz & Denzler, 2020)	WACV’20	ResNet-50	97.20
PMA$^\dagger$ (Song et al., 2020)	TIP’20	VGG16	97.40
MCL$^{*}$ (Chang et al., 2020)	TIP’20	Bilinear CNN	97.70
CAP (Behera et al., 2021)	AAAI’21	Xception	97.70
SRGNN (Bera et al., 2022)	TIP’22	Xception	97.90
Base CNN		Xception	91.90
I2-HOFI (Ours)		ResNet-50	98.69
I2-HOFI (Ours)		Xception	**99**.**00**

$^{*}$Signifies joint learning from multiple datasets to enhance accuracy

$^\dagger$Uses additional textual description

**Table 6:** Comparison of different SotA methods on NABirds (Van Horn et al., 2015) dataset

Method	Publication venue	Backbone	Accuracy (%)
SPA (Ali et al., 2019)	TIP’19	Parametric	87.60
CSPE (Korsch et al., 2019)	GCPR’19	Inception-v3	88.50
MGE (Zhang et al., 2019)	ICCV’19	ResNet-101	88.60
GaRD (Zhao et al., 2021)	CVPR’21	ResNet-50	88.00
APIN (Zhuang et al., 2020)	AAAI’21	DenseNet-161	88.10
ViT$^{\S }$ (Dosovitskiy et al., 2021)	ICLR’21	ViT-B	89.90
CAP (Behera et al., 2021)	AAAI’21	Xception	91.00
TrnFG$^{\S }$ (He et al., 2021)	AAAI’22	ViT-B	90.80
SRGNN (Bera et al., 2022)	TIP’22	Xception	91.20
RP (Chaudhuri et al., 2022)	NeurIPS’22	ResNet-50	91.20
GDSMP (Ke et al., 2023)	PR’23	ResNet-50	89.00
FET$^{\S }$ (Chen et al., 2024)	PR’24	SwinT-B	91.70
MGFF$^{\S }$ (Xu et al., 2024)	PR’24	SwinT-B	92.08
Base CNN		Xception	68.10
I2-HOFI (Ours)		ResNet-50	90.98
I2-HOFI (Ours)		Xception	**92**.**82**

$^{\S }$Denotes transformer-based approaches

### Experimental Settings

Pre-trained ImageNet weights initialize the base CNN with an image size of 256$\times$256. Data augmentation includes random rotation (±15 degrees), random scaling (1±0.15), and random cropping, resulting in a final image size of 224$\times$224. Stochastic Gradient Descent (SGD) optimizes the categorical cross-entropy loss function, starting with an initial learning rate of $10^{-2}$ and decreasing by a factor of 0.1 after every 50 epochs. The model undergoes training for 150 epochs with a mini-batch size of 8, utilizing Quadro RTX 8000 (46GB).

### Performance Comparisons with State-of-the-Art Methods

In this section, we assess our method against several SotA approaches evaluated on benchmarked FGVC datasets, as outlined in Tables 2, 3, 4, 5 and 6. For each dataset, we have chosen the top SotA methods to compare with our I2-HOFI. A detailed discussion of these comparisons will follow in the subsequent subsection.

#### Performance on Aircraft (Maji et al., 2013)

In the comparative analysis of methodologies (refer to Table 2) on this dataset, our proposed I2-HOFI model emerges as the leader with a commanding top-1 accuracy of 96.42%. This is a substantial improvement over the conventional baseline, an Xception backbone CNN, which lags behind at 79.50% accuracy. The I2-HOFI model’s superiority is further evidenced when compared to models employing the Swin Transformer-B (SwinT-B) backbone, such as CAMF and FET, which achieve 93.30% and 95.30% accuracy, respectively. While transformer-based backbones like SwinT-B are known for their high capacity and extensive parameters, making them powerful but computationally intensive, the I2-HOFI model’s efficiency stands out, suggesting an architecture that balances performance with computational pragmatism. Furthermore, the I2-HOFI notably surpasses other ResNet-50 based methods, including APCN and CAP, which record accuracies of 94.10% and 94.90%, respectively. Even more advanced models like SRGNN with Xception at 94.40% and those leveraging DenseNet-161 like APIN at 93.90% fall short of I2-HOFI’s achievement. The results demonstrate not just the raw accuracy of the I2-HOFI model but its capability to deliver SotA performance potentially with fewer parameters, a critical advantage in practical applications where model deployment efficiency is as important as accuracy.

#### Performance on CUB-200 (Wah et al., 2011)

Upon examining the results for CUB-200 dataset in Table 3, the I2-HOFI model registers a commendable accuracy of 91.60%. While this is a significant improvement over the baseline Xception CNN, which scores 75.60%, it does not clinch the highest accuracy when compared to some of the most recent methods. Notably, the FET method, which employs the transformer-based (denoted by symbol $^{\S }$) SwinT-B backbone achieves the highest accuracy of 92.90%. I2-HOFI also trails slightly behind MGFF, another SwinT-B based model, with an accuracy of 92.66%. These transformer models, while powerful, are known for their extensive parameters and computational demands. The MetaFormer-I backbone, represented by SRGN, outperforms the I2-HOFI with a 92.50% accuracy, indicating that meta-learning frameworks are also highly effective for this dataset. Moreover, the I2-HOFI model outperforms other advanced approaches, including CAP with an Xception backbone at 91.80% and TrnFG with a ViT-B at 91.70%. The comparison underlines the efficacy of I2-HOFI in utilizing a balanced architecture that possibly circumvents the computational intensity of transformers while still achieving near-top accuracy, marking it as a strong candidate for efficient and effective FGVC on the CUB-200 dataset.

#### Performance on Cars (Krause et al., 2013)

Table 4 showcases impressive performance of our I2-HOFI model on the Cars dataset, leading with an accuracy of 96.92%. It narrowly surpasses the SRGNN model which utilizes an Xception backbone and achieves an accuracy of 96.10%. I2-HOFI’s success is also notable against transformer-based (denoted by symbol ${\S }$) models such as TrnFG with a ViT-B backbone and MGFF with SwinT-B, which score 94.80% and 93.33% respectively, demonstrating that advanced transformer architectures, do not always guarantee superior performance. Furthermore, I2-HOFI significantly outperforms the baseline Xception CNN’s 84.80% accuracy, highlighting the advancements made in model architecture and training methodologies. Even against traditional backbones like ResNet-50, used in methods like S3Ns, GaRD, and PMG with accuracies in the 95% range, I2-HOFI demonstrates a clear advantage, emphasizing its robustness and optimization for the nuanced task of car recognition. This comparison underscores not only the high accuracy of the I2-HOFI model but also its efficiency, considering that transformer-based models, despite their potential for high performance, often come with increased computational costs due to their large number of parameters. The I2-HOFI model’s leading performance on the Cars dataset establishes it as a highly competitive and computationally efficient model for FGVC challenges.

**Table 7:** Computation complexity analysis of our I2-HOFI model with other SotA approaches

Method	Backbone	Datasets				Parm (GFLOPs)
		CUB	Car	NAB	Air	
Swin (Liu et al., 2021)	Swin	89.7	94.2	–	91.0	88.0 (15.4)
TASN (Zheng et al., 2019)	Resnet-50	87.0	93.8	–	–	37.3 (21.9)
MRDMN-L (Xu et al., 2021)	Resnet-50	88.6	94.2	–	–	51.2 (14.0)
CAP (Behera et al., 2021)	Xcep	91.8	95.7	–	94.1	34.2 (10.2)
SR-GNN (Bera et al., 2022)	Xcep	91.9	96.1	91.2	95.4	30.9 (**9**.**8**)
FET (Chen et al., 2024)	Swin	**92**.**9**	95.9	91.7	95.3	87.0 (26.4)
**I2-HOFI**	Xcep	91.6	**96.9**	**92**.**8**	**96**.**4**	**22**.**6** (14.2)

Trainable Parameters (Param) are in millions (M) and GFLOPs are in billions (B). Best are shown in bold

#### Performance on Oxford Flowers (Nilsback & Zisserman, 2008)

The I2-HOFI model’s performance on the Flowers101 dataset (refer Table 5) is outstanding, achieving an exceptional accuracy of 99.0%. This impressive score sets a new standard, eclipsing the baseline Xception CNN’s accuracy of 91.90% by a substantial margin. Notably, I2-HOFI surpasses advanced methods employing transfer/joint learning strategies (indicated by  in Table 5). These strategies involve leveraging knowledge from multiple datasets to improve performance on target tasks. For instance, methods such as SJFT, OPAM, and DSTL, which use ResNet-152, VGGNet, and Inception-v3 backbones respectively, and incorporate transfer/joint learning strategies, achieve accuracies ranging from 97.00 to 97.60%. I2-HOFI outperforms these methods, suggesting its superior ability to integrate and generalize knowledge across datasets for fine-grained classification. Methods like PMA with an accuracy of 97.40% incorporate additional text descriptions (denoted by $\dagger$) into their learning process. This technique offers a richer contextual understanding, allowing the model to learn from both visual features and descriptive text data, potentially leading to a more robust classifier. However, despite such sophisticated adaptation, I2-HOFI outperforms them without necessarily relying on such strategies. Even compared to the SRGNN model, which holds the second-highest accuracy of 97.90% without the indication of employing such strategies, I2-HOFI demonstrates a clear edge. This advantage highlights the model’s robustness and the effectiveness of its architecture, possibly indicating an efficient use of parameters and computational resources while still delivering top-tier performance.

#### Performance on NABirds (Van Horn et al., 2015)

On this dataset, the I2-HOFI model achieves an accuracy of 92.82% (Table 6), positioning it as the front-runner among the various SotA methods. It eclipses the baseline Xception CNN, which registers a significantly lower accuracy of 68.10%, highlighting the effectiveness of the I2-HOFI approach. Transformer-based (denoted by ${\S }$) models, such as ViT and MGFF with SwinT-B, which are typically associated with high parameter counts and computational demands, demonstrate accuracies of 89.90% and 92.08% respectively. This places I2-HOFI ahead, even as it presumably operates without the same level of computational complexity. Comparatively, I2-HOFI also outperforms traditional convolutional architectures like ResNet-50 used in GaRD and RP, which show accuracies of 88.00% and 91.20%. The DenseNet-161 backbone, utilized in APIN, lags slightly behind with an accuracy of 88.10%. CAP, employing Xception, reaches 91.00%, further showcasing that even well-established CNN architectures can fall short of the performance achieved by I2-HOFI. This performance spectrum on the NABirds dataset underscores the capability of I2-HOFI to effectively capture and classify the diverse avian species, setting it apart as an efficient and robust model for FGVC tasks.

### Model Capacity and Complexity Analysis

The computational complexity analysis of the I2-HOFI model showcases an optimal balance of efficiency and high performance. With 22.6 million parameters and 14.2 GFLOPs, I2-HOFI secures commendable accuracy across several datasets: 91.6% for CUB-200, a standout 96.9% for Cars, 92.8% for NABirds, and an exceptional 96.4% for the Aircraft dataset. Comparatively, the Swin Transformer requires substantially more resources, with 88 million parameters and 15.4 GFLOPs, yet achieves a lower 94.2% on Cars and 91.0% on Aircraft (Table 7). The FET model, also based on the Swin Transformer, demonstrates high accuracy but incurs a significant computational cost of 87.0 million parameters and 26.4 GFLOPs. On the other hand, SR-GNN, which employs an Xception backbone similar to I2-HOFI, has a comparable GFLOPs count but fewer parameters, and it slightly trails behind I2-HOFI in CUB-200 and NABirds accuracies, while it marginally surpasses I2-HOFI in Aircraft accuracy. Meanwhile, CAP, with a more modest parameter count and Xception backbone, does not quite achieve the high accuracies demonstrated by I2-HOFI. Overall, I2-HOFI not only achieves high accuracy but also distinguishes itself with computational efficiency, rendering it a potent model for applications where both precision and cost-effectiveness are pivotal.

## Ablation Study

The ablation study methodically examines several key aspects of our model’s design. We begin by assessing the combined effects of APPNP and GAT mechanisms with various graph weaving techniques on the Aircraft and Cars datasets. This is followed by an analysis of shared versus unshared GNN layers in conjunction with residual connections for Inter- and Intra-Weaving mechanisms. Next, we investigate the impact of Residual APPNP’s teleport probability $\gamma$ and the number of heads in the graph attention (GAT) mechanism, keeping other parameters fixed. Finally, we explore the influence of the partition parameter *k* related to the channel dimension $C'$, with fixed values of $\gamma$ and GAT heads. Each section of this study provides crucial insights into the configuration and efficacy of the model.

**Table 8:** Effect of various configurations on the validation accuracy (%) for the Aircraft and Cars datasets in an ablation study

Feature weaving	Residual GNN	Aircraft	Cars
Inter	APPNP	94.86	94.68
	GAT	87.05	93.05
	APPNP + GAT	91.43	93.06
Intra	APPNP	94.71	95.06
	GAT	94.92	95.63
	APPNP + GAT	94.68	94.95
Inter + Intra	APPNP	94.95	94.70
	GAT	95.58	96.17
**I2-HOFI** (Inter + Intra +APPNP + GAT)		**96**.**42**	**96**.**92**

It compares different combinations of Residual APPNP, Residual GAT, and inter- and intra-settings

Best results are shown in bold

### Implications of Graph Weaving Over GNN Mechanisms

**Fig. 6:**
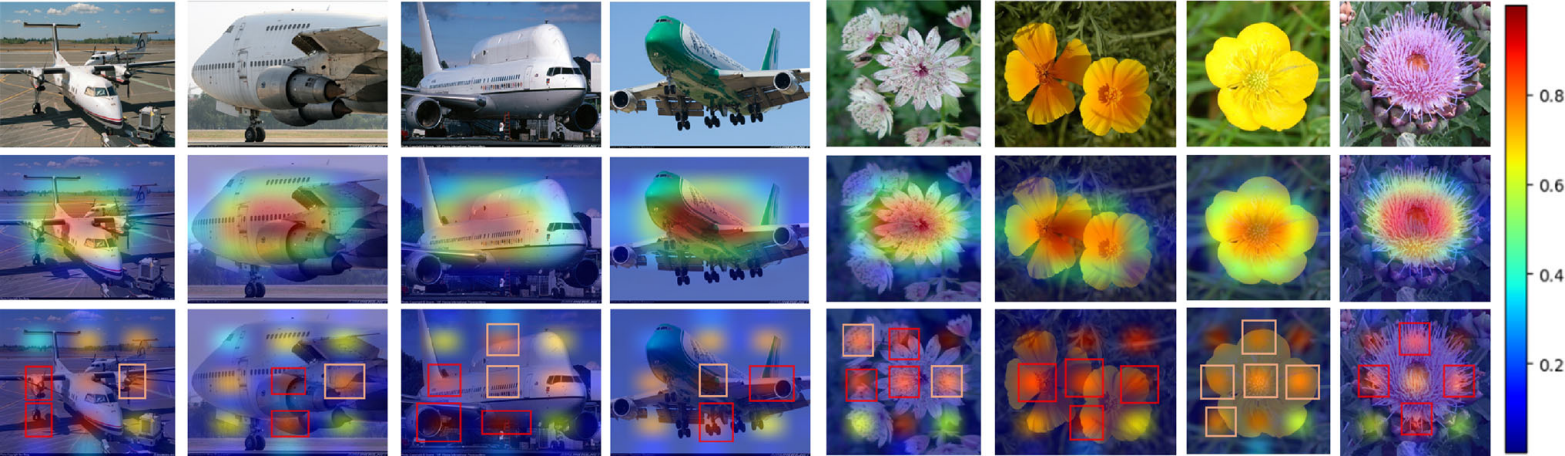
Heatmap visualizations from the Xception backbone, computed using the CAM approach. The middle row shows results from the standalone base Xception, while the bottom row shows the Xception output trained within our I2-HOFI framework, with bounding boxes further emphasizing the key regions contributing to classification

Table 8 provides a comprehensive view of how different configurations affect the I2-HOFI model’s validation accuracy on the Aircraft and Cars datasets. Notably, the model with the Inter feature weaving and residual APPNP achieves an accuracy of 94.95% on Aircraft, slightly edging out the Intra feature weaving with residual APPNP, which scores 94.71%. Conversely, for Cars, the Intra feature weaving and residual GAT combination excel with an accuracy of 95.63%, surpassing the Inter feature weaving with residual GAT at 95.58%. The combinations of residual APPNP with residual GAT yield 94.68% on Aircraft and 94.95% on Cars when using the Intra feature weaving, while the Inter feature weaving with the same GNN framework drops to 91.43% on Aircraft and rises to 93.06% on Cars. This suggests that Intra-weaving may be more critical for the Aircraft dataset, while Inter-weaving offers some advantages for the Cars dataset when combined with the dual GNN approach. The I2-HOFI model, which integrates both Inter and Intra feature weaving with the combined power of residual APPNP and residual GAT, achieves the highest accuracies of 96.42% for Aircraft and 96.92% for Cars. These results indicate that the full configuration leverages the strengths of each component, ensuring a comprehensive feature analysis that captures both the detailed nuances and broader contextual patterns essential for fine-grained classification (see Fig. 6). The residual connections in both APPNP and GAT likely contribute to this performance by enabling effective feature propagation and attention-based weighting without information loss, demonstrating the model’s prowess in complex visual recognition tasks. It is noted that too many layers can blur the distinction between inter-ROI and intra-ROI graphs due to over-smoothing. To address this, we use residual connections to preserve initial node features and stabilize training, carefully manage the power iteration step in APPNP to prevent performance degradation, and employ GAT’s attention mechanisms to emphasize critical features. Additionally, weaving intra-ROI and inter-ROI graphs processed with shared GNN weights balances and maintains their distinction. These strategies ensure our model preserves distinctive features for FGVC while mitigating over-smoothing risks.

**Table 9:** Accuracy (%) with different key variations in the architecture

Key modules	Datasets			
	Aircraft	Cars	Flowers	NABirds
W/o Residual + Unshared	96.1	91.9	97.3	84.7
W/o Residual + Shared	95.3	96.1	97.4	86.6
Residual + Unshared	94.3	96.4	98.8	91.7
Residual + Shared (**I2-HOFI**)	**96**.**4**	**96**.**9**	**99**.**0**	**92.8**

We experimented with varying combinations of with and without residual connection on APPNP and GAT along with either shared weight parameter set between the Inter and Intra ROI graphs

Best results are shown in bold

### Impact of Weight Sharing and Residuals in GNNs

Table 9 presents an ablation study to evaluate the impact of shared versus unshared weights and the presence of residual connections in the model’s architecture across four different datasets: Aircraft, Cars, Flowers, and NABirds. The results indicate that models without residual connections but with unshared weights achieve an accuracy of 96.1% on Aircraft and 91.9% on Cars, showing that distinct feature transformations for different graph weavings can effectively capture domain-specific nuances without the need for residual learning. However, when the weights are shared between the inter and intra graphs without residual connections, there is a slight decrease in performance for Aircraft to 95.3%, suggesting that unshared weights are more beneficial for this dataset. For Cars, the shared weights configuration without residuals slightly improves performance to 96.1%, indicating that sharing weights may be beneficial in cases where similar features are indicative of the class labels across different regions of the image. In contrast, when residual connections are introduced, the configuration of the unshared weight underperforms for Aircraft with an accuracy of 94.3%, suggesting that the additional complexity of residuals may require more specialized transformations that unshared weights can provide. On the other hand, the residual model with unshared weights significantly improves the accuracy for Cars to 96.4%, outperforming the non-residual counterpart and indicating the positive impact of residuals in conjunction with unshared weights for this dataset. The final configuration, which is the I2-HOFI model itself, uses both residual connections and shared weights. This configuration yields the best accuracy for Aircraft at 96.4% and for Cars at 96.9%. It also performs exceptionally well on the Flowers and NABirds datasets with accuracies of 99.0% and 92.8%, respectively. This demonstrates that the synergistic combination of residuals and shared weights across inter and intra graphs can lead to superior performance by efficiently leveraging information from both local and global feature interactions while mitigating overfitting and enhancing generalization. These results suggest that the I2-HOFI model’s architecture, which combines residual learning with shared weights across its graph weaving mechanisms, is robust and adaptable across various FGVC tasks, successfully capturing both the detailed and global discriminative features needed for high accuracy.

**Table 10:** Impact of varying hyperparameters of APPNP and GAT in GNN

Dataset	Varying $\gamma$ values in APPNP								Changing #Heads in GAT				
	0.1	0.2	0.3	0.4	0.5	0.6	0.7	0.8	1	2	4	6	8
Aircraft	95.76	95.52	**96**.**42**	96.12	95.28	95.16	94.59	95.22	**96**.**42**	94.86	94.50	94.71	94.62
Cars	96.19	95.97	**96**.**92**	95.97	96.08	96.16	96.09	95.97	**96**.**92**	94.95	94.46	95.07	94.66
Flowers	97.80	98.71	98.77	98.68	98.58	98.88	98.67	**99**.**00**	**98**.**77**	97.41	97.54	97.43	97.43
NABirds	86.73	91.40	91.38	91.63	91.67	91.77	91.64	**91**.**82**	91.38	91.61	91.80	**91**.**82**	91.53

This ablation is done by keeping the other parameters *k* fixed at 2 (or corresponding node feature dimension $C'$ fixed to 512). Also parameter $\gamma$ and #Heads in GAT are varied by alternately fixing the other parameter

Best results are shown in bold

### Impact of APPNP’s Teleport Probability ($\gamma$) and Graph Attention Heads

The ablation study outlined in Table 10 conducts an in-depth analysis of the impact of key hyperparameters within the GNN framework of our I2-HOFI model. The study methodically varies the teleport probability $\gamma$ in the APPNP propagation algorithm and the number of attention heads in the graph attention (GAT) mechanism, while maintaining constant the other parameters, notably $k=2$ and the node feature dimension $C'=512$. This approach aims to determine the optimal settings for $\gamma$ and the number of attention heads, providing a comprehensive examination by adjusting $\gamma$ from 0.1 to 0.8 and the number of GAT heads from 1 to 8. For the Aircraft dataset, the optimal accuracy of 96.42% is observed when $\gamma =0.3$ and the GAT has 1 head, suggesting a balanced utilization of local and root node features. This configuration also proves to be effective for the Cars dataset, achieving peak accuracy at 96.92% under the same $\gamma$ and GAT head conditions, supporting the notion that moderate feature propagation and a streamlined attention mechanism are adequate for capturing dataset-specific patterns. The Flowers dataset displays high accuracy, peaking close to 99.0% and 98.77%, across varying $\gamma$ values and GAT head counts, indicating a lower sensitivity to these hyperparameters. This could be attributed to the dataset’s characteristics, where features are possibly less complex or more distinct, thus requiring minimal hyperparameter adjustments. Conversely, the NABirds dataset shows an incremental improvement in accuracy with increasing $\gamma$, reaching its highest at $\gamma =0.8$ with 91.82%. This trend reveals the importance of global information for the model’s performance in this particular dataset and suggests that the dataset might possess high inter-class separation, allowing for more aggressive feature mixing. The number of GAT heads also varied, with the NABirds dataset showing the highest accuracy at 6 heads with 91.82%. This outcome implies that the model efficiently captures feature patterns without overcomplicating the attention mechanism, hence reducing the necessity for a large number of attention heads. In summary, the I2-HOFI model demonstrates that achieving high accuracy in the majority of the datasets is possible with lower $\gamma$ values in APPNP and fewer heads in GAT. This indicates that the model proficiently captures and utilizes local contextual information and can distill essential information for fine-grained classification tasks without the added complexity of numerous attention heads.

### Impact of Node Feature Dimensionality $C'$

Table 11 examines the impact of node feature dimensionality on classification performance by altering the partition parameter *k* for the channel dimension $C'$, thereby adjusting the granularity of the feature representation across four distinct datasets. Each column denotes $2^k$ partitions, effectively scaling the dimensionality of the node features processed by the network. To isolate the effects of *k*, the teleport probability $\gamma$ and the number of heads in GAT are maintained at 0.3 and 1, respectively, in line with optimal values identified in Table 10. In the Aircraft dataset, an intermediate level of partitioning with $k = 2$ (512 features) achieves the highest accuracy of 96.42%, suggesting that a balance between feature granularity and model complexity is key for effective discrimination. The Cars dataset mirrors this observation, with $k = 2$ also delivering the best performing accuracy of 96.92%, reinforcing the notion that a moderate partition size is ideal for accurate feature extraction without the risk of overfitting. For Flowers, the peak accuracy of 99.0% is attained at $k = 2$ underscores a similar preference for a mid-range feature dimensionality, possibly due to the unique visual characteristics of flowers that can be captured without excessively fine or broad feature partitions. Conversely, the NABirds dataset deviates from this trend, achieving its best accuracy of 92.82% at $k = 1$, corresponding to 1024 features. This implies that the intricate variations within avian species require a larger feature set for the model to perform optimal classification. Collectively, these findings from Table 11 suggest that the FGVC task benefits significantly from carefully calibrated node feature dimensionality, which hinges on the unique complexities and distinctiveness of features within each specific dataset. Selecting an appropriate value of *k* is critical for the model to effectively discern discriminative features while maintaining computational efficiency and avoiding overfitting.

### Interplay Between Number and Size of ROIs

**Fig. 7:**
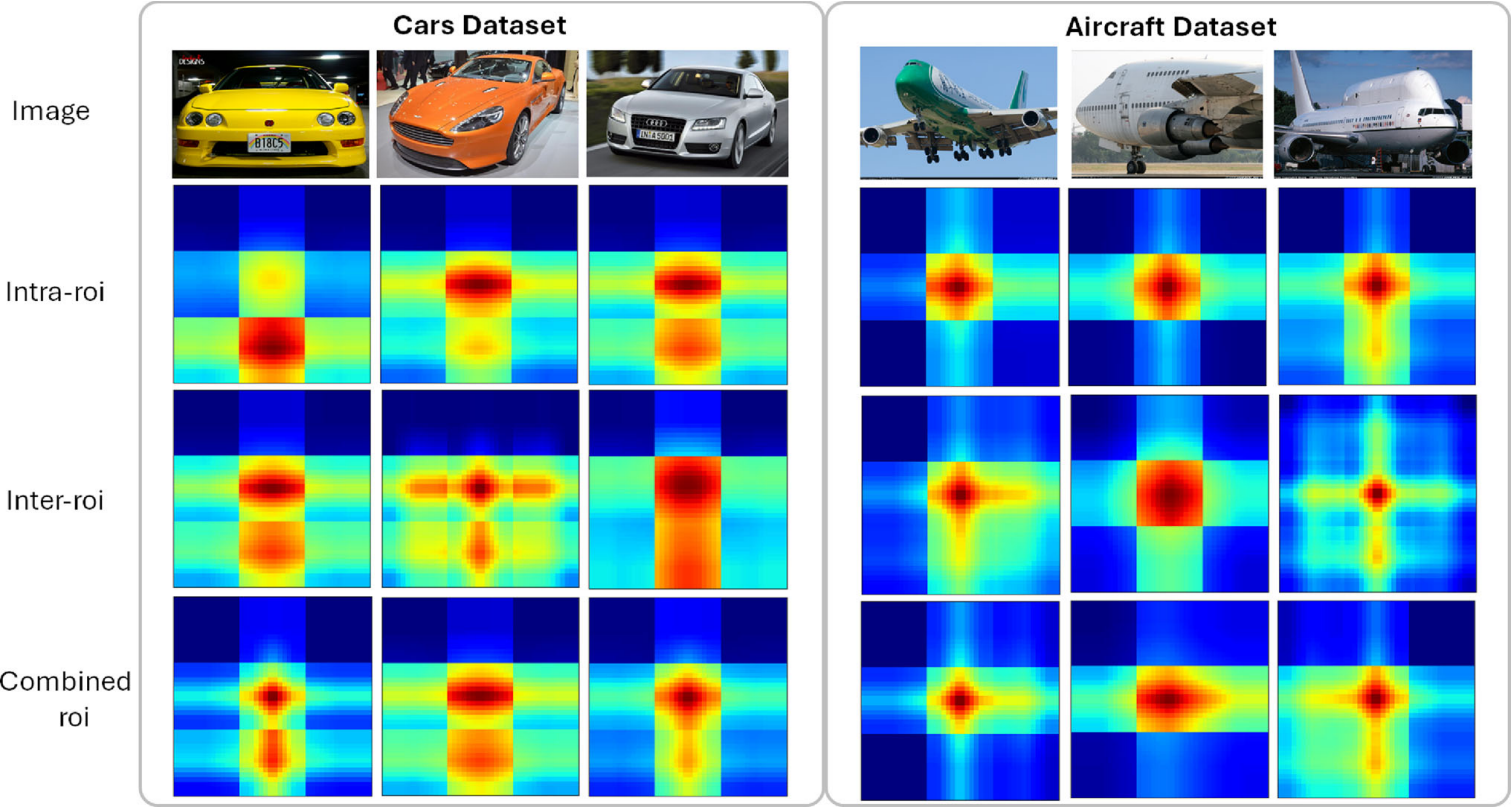
Top 5 ROIs selected based on maximum mean activation. Heatmaps are computed for each region using the CAM approach and overlaid on a 42 $\times$ 42 background, corresponding to the original ROI regions. The top 5 ROIs are then summed and visualized for aircraft and car images, showing intra-ROI, inter-ROI, and combined-ROI activations

**Fig. 8:**
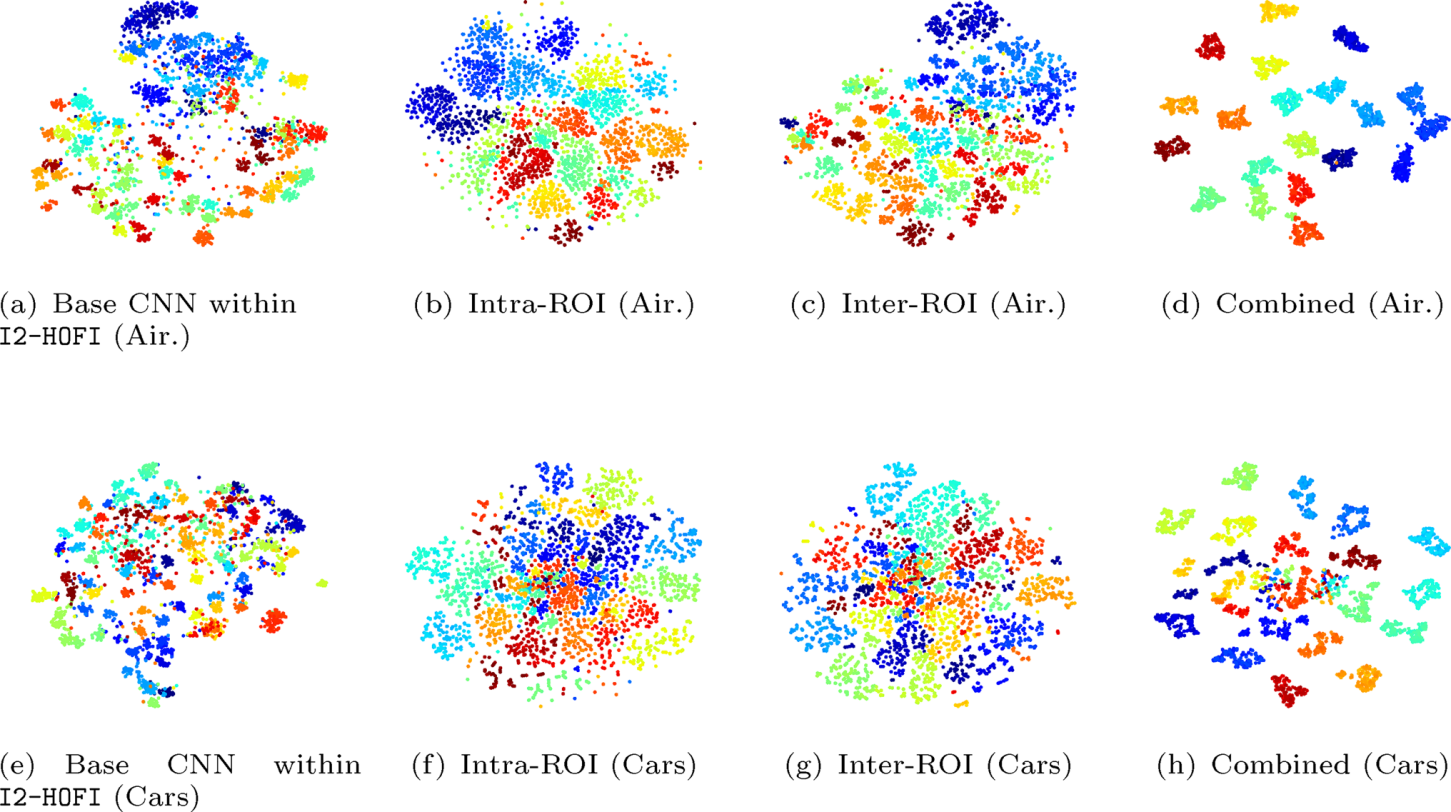
t-SNE visualizations of I2-HOFI feature embeddings for Aircraft (top) and Cars (bottom), showing progression through **a**, **e** Base CNN (Xception) backbone, **b**, **f** intra-ROI, **c**, **g** inter-ROI, and **d**, **h** combined through gated attention pooling (Fig. 2). The plots highlight improved class separation and clustering with GNN-based region interactions

Table 12 elaborates on comprehensive experiments illustrating the effects of the interplay between the number and size of ROIs on our model’s accuracy across different datasets. From our experiments, we found that increasing the number of ROIs from 11 to 27 when the ROI size is 3 $\times$ 3 results in a significant accuracy improvement from 96.1 to 96.9% in the Cars dataset. However, further increasing the number of ROIs to 36 and 64 leads to a slight decrease in accuracy to 95.8% and 95.6%, respectively. This indicates an optimal point around 27 ROIs for the 3 $\times$ 3 size. A similar pattern is observed for the Aircraft dataset, where the highest accuracy (96.4%) is achieved with 27 ROIs of size 3 $\times$ 3. For the Flowers dataset, increasing the number of ROIs from 11 to 27 yields the highest accuracy (99.0%), with a slight decrease as the number of ROIs continues to increase. For the CUB200 dataset, the accuracy remains relatively stable between 91.4 and 91.6% for varying numbers of ROIs with 3 $\times$ 3 size, indicating a lesser sensitivity to the number of ROIs. When using a larger ROI size of $5 \times 5$, the impact of increasing the number of ROIs shows a different trend. For the Cars dataset, accuracy peaks at 95.8% with 27 ROIs but drops more significantly as the number of ROIs increases to 64 (93.4%). The Aircraft dataset shows high accuracy (96.0%) with 11 ROIs of size 5x5, but a marked decrease when the number of ROIs increases. For the Flowers dataset, the highest accuracy (98.8%) is observed with 11 ROIs of size 5x5. Increasing the number of ROIs leads to a gradual decrease in accuracy. The CUB200 dataset follows a similar trend, where fewer, larger ROIs (11 of size 5x5) provide better accuracy compared to a higher number of ROIs. We have provided a visualization in Fig. 7 that illustrates how the top 5 ROIs, based on mean activation, emphasize the most relevant regions for classification. Additionally, Fig. 8 presents t-SNE visualizations of the feature embeddings, showing how the model’s region interactions contribute to improved class separation and clustering across datasets.

**Table 11:** Effect of varying partition parameter *k* on accuracy across datasets, with each column representing $2^k$ partitioned channel dimension $C'$

Dataset	Varing integer parameter $2^k$			
	$2^1$ (1024)	$2^2$ (512)	$2^3$ (256)	$2^4$ (128)
Aircraft	95.19	**96**.**42**	95.67	94.92
Cars	95.81	**96**.**92**	96.01	94.83
Flowers	98.23	**99**.**00**	98.32	97.35
NABirds	**92**.**82**	91.38	91.28	90.47

Best results are shown in bold

**Table 12:** Interplay between the number of ROIs (nROI) and size of ROIs (sROI) and their respective accuracy on different benchmark datasets

sROI	nROI	Datasets			
		Cars	Aircraft	Flowers	CUB200
3 $\times$ 3	11	96.1	95.3	98.4	91.5
	27	**96**.**9**	**96**.**4**	**99**.**0**	**91**.**6**
	36	95.8	94.9	97.4	91.5
	64	95.6	95.2	98.3	91.4
$5\times 5$	11	95.5	96.0	97.7	90.5
	27	95.8	91.5	97.5	89.1
	36	95.4	94.5	97.4	90.0
	64	93.4	93.3	96.4	86.5

Best results are shown in bold

Overall, our experiments clearly show that there is an optimal number of ROIs for a given size that maximizes performance. Specifically, 27 ROIs of size 3 $\times$ 3 generally provide the best balance for most datasets. This configuration effectively captures the necessary local features for FGVC without overwhelming the GNN’s capacity with excessive information. When the number of ROIs is too low (e.g., 11 ROIs), the model may not capture sufficient local detail, leading to suboptimal performance. Conversely, an excessive number of ROIs (e.g., 64 ROIs) can lead to over-segmentation, where the model must manage too many fine details. This increases computational complexity and the risk of overfitting, as the model might focus on irrelevant or redundant information, degrading performance. We also observe that the size of ROIs impacts different datasets differently. In datasets like Aircraft and Cars, with high intra-class variation, setting an optimal number of ROIs is more critical than in datasets like Flowers, which naturally capture more distinctive features and are less sensitive to the number of ROIs in achieving optimal performance. These findings validate our approach and illustrate the importance of carefully tuning the number and size of ROIs to achieve optimal performance. The experiments highlight that while more ROIs can capture more local features, there is a point where too many ROIs can lead to diminishing returns or even degrade performance, likely due to increased complexity and potential for overfitting.

### Impact of Different Backbone Architecture

We conducted a comprehensive set of experiments using multiple backbone architectures (including lightweight models) to evaluate the effectiveness of our **I2-HOFI** enhancement as described in Table 13. Specifically, we categorized the backbones into small (lightweight) and large types and conducted experiments on the base models by simply replacing the last activation layer to correspond to the number of classes, versus adding our **I2-HOFI** GNN architecture. This approach ensures a fair comparison and highlights the robustness and general applicability of our method across different backbone architectures. Additionally, we performed an ablation study with ResNet50 and Xception backbones on the Aircraft and Cars datasets to compare performance at different image resolutions (224 $\times$ 224 vs. 448 $\times$ 448) (see Table 14). While increasing the resolution to 448x448 improves accuracy, the computational cost (GFLOPs) rises significantly. For instance, with ResNet50 backbone GFLOPs increase from 12.9 to 36.0 B, yielding only marginal accuracy gains. This highlights the efficiency of our **I2-HOFI** framework with lighter backbones like Xception, which achieves superior performance with fewer parameters and lower GFLOPs at 224 $\times$ 224 resolution.

**Table 13:** Performance and computational complexity of various backbone type with and without the our **I2-HOFI** enhancement on different datasets

Backbone size	Methods	Datasets			Param (GFLOPs)
		Cars	Aircraft	Flowers	
Small	MobNetV3 (base) (Howard et al., 2019)	79.96	68.86	88.12	**4**.**2** (**0**.**5**)
	MobNetV3 + **I2-HOFI**	93.97	90.43	96.35	5.8 (3.1)
	NasNet-M (base) (Zoph et al., 2018)	77.86	70.45	87.54	5.6 (1.3)
	NasNet-M + **I2-HOFI**	94.59	93.08	98.37	7.1 (3.8)
	DenseNet121 (base) (Huang et al., 2017)	86.67	80.41	92.95	7.2 (5.7)
	DenseNet121 + **I2-HOFI**	93.98	94.04	98.89	8.8 (8.2)
Large	ResNet50 (base) (He et al., 2016)	85.79	80.25	92.23	24.0 (7.8)
	ResNet50 + **I2-HOFI**	94.33	92.26	98.69	25.4 (12.9)
	Xception (base) (Chollet, 2017)	84.80	79.50	91.90	21.3 (9.1)
	Xception + **I2-HOFI**	**96**.**90**	**96**.**40**	**99**.**00**	22.6 (14.2)

Trainable parameters (Param) are in millions (M) and GFLOPs are in billions (B). Best results are shown in bold

**Table 14:** Accuracy (%) of our I2-HOFI model with ResNet50 and Xception backbones on Aircraft and Cars datasets with different input image sizes

Backbone	224$\times$224 size			448$\times$448 size			Param
	Aircraft	Cars	GFLOPS	Aircraft	Cars	GFLOPS	
ResNet50	92.3	94.3	**12**.**9**	93.8	95.6	**36**.**0**	25.4
Xception	**96**.**4**	**96**.**9**	14.2	**96**.**7**	**97**.**3**	41.7	**22**.**6**

Trainable parameters (Param) are in millions (M) and GFLOPs are in billions (B). Best results are shown in bold

With smaller backbones like the recently released MobNetV3 (Howard et al., 2019), our **I2-HOFI** enhancement significantly improves performance, achieving 93.97% accuracy on Cars and 96.35% on Flowers. This represents a substantial gain over the base model, which has accuracies of 79.96% and 88.12% on Cars and Flowers, respectively, while maintaining a low computational footprint (5.8 million parameters and 3.1 GFLOPS). For NasNet-M (Zoph et al., 2018), the base model’s performance on Cars is 77.86%, which increases to 94.59% with **I2-HOFI**. Similarly, for Flowers, accuracy rises from 87.54 to 98.37%, showcasing the effectiveness of **I2-HOFI** across different lightweight backbones. With DenseNet121 (Huang et al., 2017), which has relatively more parameters than MobNetV3 and NasNet-M, the base model’s performance on Aircraft improves from 80.41 to 94.04% with **I2-HOFI**. This consistent improvement across datasets highlights the robustness of our **I2-HOFI**. For large models, we experimented with ResNet50 (He et al., 2016) and Xception (Chollet, 2017) as the backbones. The ResNet50 base model achieves 85.79% on Cars and 92.23% on Flowers. When integrated with **I2-HOFI**, the accuracy jumps to 94.33% on Cars and 98.69% on Flowers, illustrating the model’s ability to significantly boost performance even on larger backbones. The Xception base model shows strong performance with 84.80% on Cars and 91.90% on Flowers. When enhanced with **I2-HOFI**, the model achieves the highest accuracies across all datasets, with 96.90% on Cars, 96.40% on Aircraft, and 99.00% on Flowers, confirming the universal applicability and effectiveness of our approach.

**Fig. 9:**
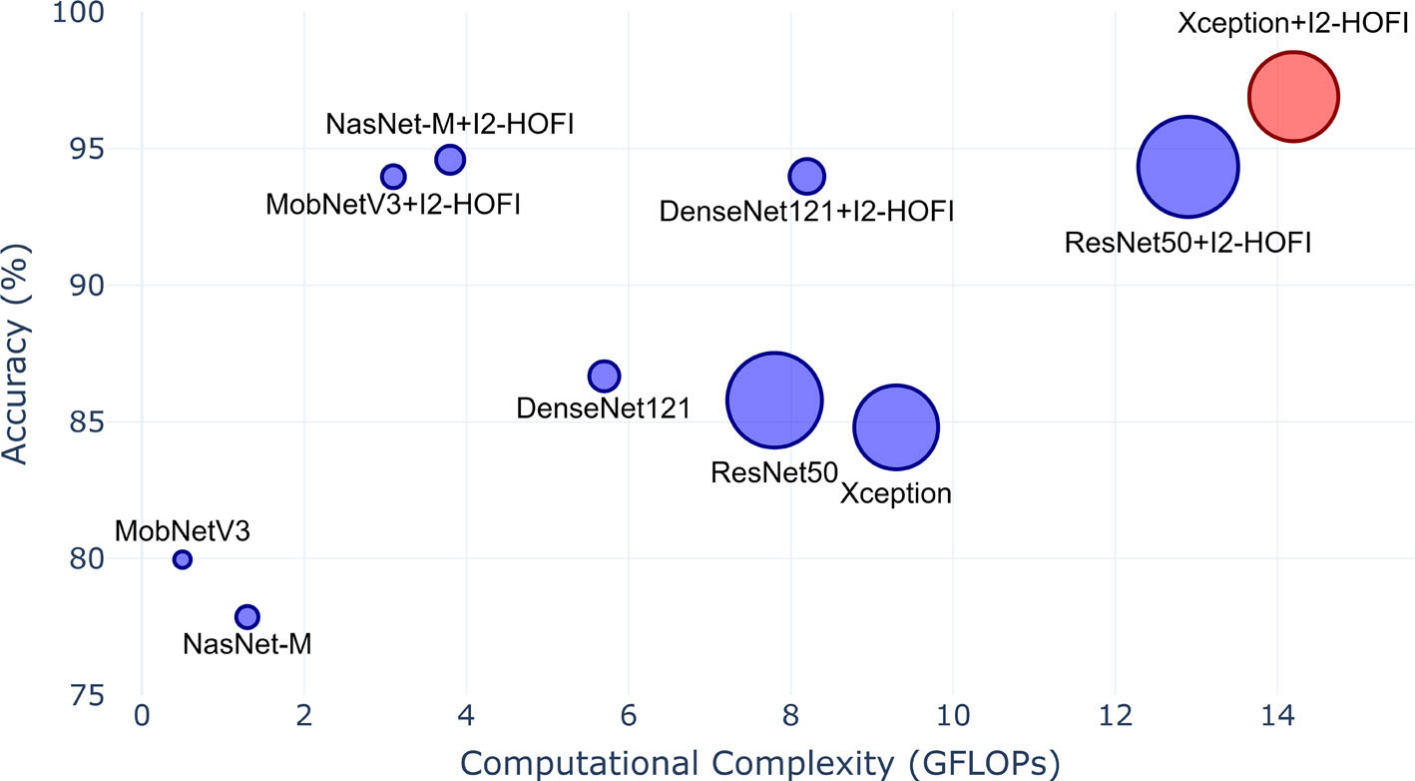
Top-1 Accuracy (%) versus computational complexity (GFLOPs) on the Cars dataset. The radius of each marker represents the model’s trainable parameter. The Xception+**I2-HOFI** model (red) achieves the highest accuracy (96.90%) with 14.2 GFLOPs (Color figure online)

In addition to this comparison, we also computed each model’s total trainable parameters along with Giga-Floating point operations (GFLOPS) to evaluate the efficacy of integrating **I2-HOFI** with the backbones. While the trainable parameters and GFLOPS increase with the **I2-HOFI** enhancement, the trade-off is justified by the significant gain in accuracy. Specifically, for lightweight backbones like MobNetV3 (Howard et al., 2019) and NasNet-M (Zoph et al., 2018), the **I2-HOFI** enhancement leads to a substantial improvement in accuracy while maintaining a modest increase in computational demands. For instance, MobNetV3’s parameters increase from 4.2 to 5.8 million, and GFLOPS rise from 0.5 to 3.1, while accuracy on the Cars dataset improves from 79.96 to 93.97%. Similarly, for NasNet-M, parameters and GFLOPS increase from 5.6 to 7.1 million and 1.3 to 3.8, respectively, with accuracy jumping from 77.86 to 94.59%. When applied to larger backbones such as ResNet50 (He et al., 2016) and Xception (Chollet, 2017), the **I2-HOFI** enhancement results in substantial performance improvements with a moderate increase in computational complexity. ResNet50’s parameters rise from 24 to 25.4 million, and GFLOPS increase from 7.8 to 12.9, while accuracy on the Cars dataset improves from 85.79 to 94.33%. The Xception backbone, which sees parameters increase from 21.3 to 22.6 million and GFLOPS from 9.1 to 14.2, achieves the highest accuracy across all datasets, with significant improvements such as 96.90% on Cars (Fig. 9).

These results clearly demonstrate that our **I2-HOFI** enhancement consistently improves performance across various backbones, whether small or large. This indicates that our method is not dependent on a specific backbone architecture and can be effectively integrated with different types of models to achieve SotA results. The increase in computational resources is balanced by the significant performance gains, validating the efficiency and scalability of our **I2-HOFI** approach across diverse backbone architectures.

## Conclusion

This paper introduces I2-HOFI, an innovative and efficient framework designed for fine-grained visual classification. The uniqueness of I2-HOFI lies in its utilization of inter- and intra-region graph weaving mechanisms to capture intricate patterns within images. Operating within the realm of graph neural networks, our approach not only demonstrates superior performance on benchmark datasets but also accomplishes this with significantly reduced computational overhead compared to existing models. Distinguishing itself from prior fine-grained visual recognition methods that often compartmentalize global and local features, our approach seamlessly integrates both via inter- and intra-region graphs. The inter-region graphs are adept at capturing long-range dependencies, enabling the recognition of global patterns. Simultaneously, the intra-region graphs focus on the finer details within specific object regions, facilitating the capture of subtle variations. The adaptability and scalability of I2-HOFI pave the way for its application in various real-world scenarios, promising significant advancements in both the theoretical foundations and practical applications of fine-grained image recognition. I2-HOFI, with its innovative graph-based mechanisms, opens new avenues for pushing the boundaries of what is achievable in fine-grained visual classification.

## Data Availability

The datasets used in this work are publicly available and can be downloaded from the following links: (1) Aircraft dataset (Maji et al., 2013) (*arXiv: 1306.5151* https://arxiv.org/abs/1306.5151) is accessible at: https://www.robots.ox.ac.uk/vgg/data/fgvc-aircraft/. (2) Caltech-UCSD Birds (CUB-200) dataset (Wah et al., 2011) (doi: https://doi.org/10.22002/D1.20098) can be found at: https://data.caltech.edu/records/65de6-vp158. (3) Stanford Cars dataset (Krause et al., 2013) (doi: https://doi.org/10.1109/ICCVW.2013.77) is available at: https://pytorch.org/vision/0.16/generated/torchvision.datasets.StanfordCars.html. (4) Oxford Flowers dataset (Nilsback & Zisserman, 2008) (doi: https://doi.org/10.1109/ICVGIP.2008.47) can be accessed at: https://www.robots.ox.ac.uk/vgg/data/flowers/102/. (5) NABirds dataset (Van Horn et al., 2015) (doi: https://doi.org/10.1109/CVPR.2015.7298658) is available at: https://dl.allaboutbirds.org/nabirds.
